# Selective Inhibition of the C-Domain of ACE (Angiotensin-Converting Enzyme) Combined With Inhibition of NEP (Neprilysin): A Potential New Therapy for Hypertension

**DOI:** 10.1161/HYPERTENSIONAHA.121.17041

**Published:** 2021-07-26

**Authors:** Rhéure Alves-Lopes, Augusto C. Montezano, Karla B. Neves, Adam Harvey, Francisco J. Rios, Dominik S. Skiba, Lauren B. Arendse, Tomasz J. Guzik, Delyth Graham, Marko Poglitsch, Edward Sturrock, Rhian M. Touyz

**Affiliations:** 1 Institute of Cardiovascular and Medical Sciences, University of Glasgow, United Kingdom (R.A.-L., A.C.M., K.B.N., A.H., F.J.R., D.S.S., T.J.G., D.G., R.M.T.).; 2 Attoquant Diagnostics GmbH, Vienna, Austria (M.P).; 3 Institute of Infectious Disease and Molecular Medicine and Division of Medical Biochemistry, University of Cape Town, South Africa (L.B.A., E.S.).

**Keywords:** blood pressure, neprilysin, omapatrilat, permeability, vasodilatation

## Abstract

Combined inhibition of NEP (neutral endopeptidase) and ACE (angiotensin-converting enzyme), without unwanted effects, remains an attractive therapeutic strategy in cardiovascular medicine. Omapatrilat, a dual NEP inhibitor–ACE inhibitor, was a promising antihypertensive drug but failed in trials due to angioedema, an effect possibly caused by inhibition of both the N- and C-domains of ACE. Here, we aimed to determine whether lisinopril-tryptophan (lisW-S), a C-domain specific ACE inhibitor that preserves the N-domain catalytic activity, together with sacubitril (NEP inhibitor), differentially influences cardiovascular function and vascular permeability in hypertension compared with omapatrilat and lisinopril+sacubitril which inhibits both the ACE C- and N-domains. Ang II (angiotensin II)–dependent hypertensive mice (transgenic mice expressing active human renin in the liver [also known as LinA3]) received vehicle, sacubitril, lisW-S, lisinopril, lisinopril+sacubitril, or lisW-S+sacubitril for 4 weeks. Systolic blood pressure was increased in LinA3 mice, along with cardiac hypertrophy/dysfunction, impaired endothelium-dependent vasorelaxation, hypercontractile responses, vascular remodeling, and renal inflammation. LisW-S+sacubitril, lisinopril+sacubitril, and omapatrilat reduced systolic blood pressure and normalized cardiovascular remodeling and vascular hypercontractile responses in LinA3 mice. Although lisinopril+sacubitril and omapatrilat improved Ach-induced vasorelaxation, lisW-S+sacubitril had no effect. Endothelial permeability (Evans Blue assessment) was increased in omapatrilat but not in LisW-S+sacubitril–treated mice. In conclusion, lisW-S combined with sacubitril reduced systolic blood pressure and improved cardiac dysfunction in LinA3 mice, similar to omapatrilat but without effects on endothelium-dependent vasorelaxation. Moreover, increased vascular leakage (plasma extravasation) induced by omapatrilat was not evident in mice treated with lisW-S+sacubitril. Targeting ACE C-domain and NEP as a combination therapy may be as effective as omapatrilat in lowering systolic blood pressure, but without inducing vascular permeability and endothelial injury.

## Abstract 2

Supplemental Digital Content is available in the text.

The renin-angiotensin-aldosterone system plays an important role in regulating blood pressure (BP) and is a key factor underlying the development of hypertension, with major clinical guidelines recommending inhibition of this system as one of the first-line drugs, along with other drugs including calcium channel blockers and diuretics.^1^ Clinically, this is achieved with ACE (angiotensin-converting enzyme) inhibitors and angiotensin receptor blockers.^2,3^ ACE is a zinc-dependent carboxypeptidase with 2 distinct catalytic domains, N- and C-domains, each with different enzymatic activities.^4^ The C-domain hydrolyzes Ang I (angiotensin I) and is primarily responsible for Ang II (angiotensin II) production. However, both domains contribute to degradation of BK (bradykinin), and BK degradation by either domain is sufficient to compensate for the absence of the other.^5–8^ Currently, ACE inhibitor drugs that are used clinically target both C- and N-domains. Consequently, the reduction of Ang II levels through C-domain ACE inhibition is associated with an increase in active BK, which has been implicated in ACE inhibitor–induced cough and angioedema.^9–11^ Possibly, specific inhibition of C-domain might reduce Ang II production while maintaining physiological levels of BK.

Although ACE is thought to be the primary site of BK degradation, other enzymes including NEP (neprilysin), a membrane-bound zinc-metallopeptidase most abundant in the kidneys and lungs, also contribute to BK degradation.^12,13^ NEP is involved in the degradation of many peptides besides BK, including the vasodilator and diuretic natriuretic peptides (ANP [atrial natriuretic peptide], BNP [brain natriuretic peptide], and CNP [C-type natriuretic peptide]). NEP also hydrolyzes both Ang I and Ang II.^14–16^ Initially, it was hypothesized that inhibition of the natriuretic peptide system would counter-regulate the detrimental effects of renin-angiotensin-aldosterone system upregulation in hypertension by increasing accumulation of the vasodilators BK and natriuretic peptides. However, experimental and clinical studies showed that long-term NEP inhibition did not reduce BP, possibly due to concomitant inhibition of degradation of vasoconstrictors, such as Ang II and endothelin-1.^15,17–19^ It was subsequently proposed that NEP inhibitors combined with ACE inhibitor would be more effective than inhibition of a single pathway. This led to the development of omapatrilat, a drug that inhibits both ACE and NEP.^20^ Preclinical and early clinical studies with this dual inhibition were promising showing potent antihypertensive effects with beneficial effects on cardiac function in patients with heart failure.^21,22^ The OCTAVE study (Omapatrilat Cardiovascular Treatment Assessment Versus Enalapril) confirmed the antihypertensive effects of omapatrilat; however, this was associated with significant angioedema,^23^ which halted further clinical development of these drugs.^20^ Omapatrilat-induced angioedema was attributed to the simultaneous inhibition of both the ACE and NEP pathways affecting BK degradation.^20^ This prompted the search for alternative approaches where BK would not be affected, such as combination angiotensin receptor blocker (valsartan) and NEP inhibitor (sacubitril) (LCZ696), and dual ECE (endothelin-converting enzyme)-NEP inhibitor (SLV-306 [daglutril]).^19,24,25^ Clinical trials demonstrated that sacubitril-valsartan is superior to ACE inhibitor in preventing deaths and hospitalization in patients with heart failure with reduced ejection fraction,^24,26^ but not in patients with heart failure with preserved ejection fraction,^27^ highlighting the need for further drug discovery targeting the renin-angiotensin-aldosterone system and NEP systems. We focused on a new paradigm using a C-domain–selective ACE inhibitor that would separate the effects on Ang II generation from BK degradation,^28^ in combination with a NEP inhibitor.^29^

We developed a C-domain-selective derivative of lisinopril (lisinopril-tryptophan [lisW-S]) by substituting a tryptophan for the P2’ proline.^28–30^ In TtRhRen mice (LinA3), a model of Ang II–dependent hypertension, lisW-S effectively reduced Ang II levels and lowered BP without influencing BK levels.^30^ The effect of lisW-S combined with a NEP inhibitor could have the same beneficial effects as omapatrilat but without the undesirable properties attributed to BK accumulation. We, therefore, hypothesized that this new combination would reduce BP, improve cardiovascular function, and reduce Ang II levels without affecting BK levels. To gain a better understanding of the systematic actions of this combined treatment, we compared its effects to sacubitril, lisinopril (inhibits ACE C- and N-domains), lisinopril+sacubitril, and omapatrilat (inhibits ACE C- and N-domains).

## Methods

The data that support the findings of this study are available from the corresponding author upon reasonable request.

### Mice and Drug Treatment

TTRhRen mice (LinA3) express human prorenin under the control of the transthyretin promoter.^31^ A furin cleavage site was inserted adjacent to the active renin molecule, resulting in cleavage by endogenous proteases and the production of active human renin. Male hemizygous LinA3 mice and their wild-type (WT) littermates were used on a C57BL/6 background and were aged 3 months at the start of study. For each set of experiments, 4 to 9 mice were studied. LinA3 mice have been fully characterized.^31,32^ They gradually develop hypertension with aging and recapitulate human essential hypertension.^30,32^ Animals were housed under 12-hour light/dark cycles at ambient temperature and were maintained on normal mouse chow. Experiments were approved by the University of Glasgow Animal Welfare and Ethics Review Board. All experimental protocols on mice were performed in accordance with the United Kingdom Animals Scientific Procedures Act 1986 (License No. 70/9021) and Animal Research: Reporting of In Vivo Experiments Guidelines.

Twelve-week-old WT and LinA3 transgenic mice were treated with lisW-S (3.5 mg/kg of body weight per day), lisinopril (1 mg/kg of body weight per day), sacubitril (1 mg/kg of body weight per day), omapatrilat (1 mg/kg of body weight per day) and sacubitril combined with lisW-S or lisinopril, or vehicle (ethanol/kolliphor EL/water: 10:10:80) via osmotic mini-pumps (Alzet) for a period of 4 weeks. Doses of drugs were based on previously published data.^28,30,33–36^ The mechanism of action of each drug is described in Figure S1 in the Data Supplement. Osmotic mini-pumps were inserted in anesthetized mice as we previously described. Mice were anesthetized with 5% isoflurane in 1.5 L/min O_2_, reduced to 2.5% isoflurane (1.5 L/min O_2_) to maintain anesthesia during the procedure. For postoperative analgesia, 10.0 mg/kg carprofen was injected subcutaneously.

### BP Measurement and Echocardiography

Systolic BP was assessed by tailcuff plethysmography (Visitech Systems model BP-2000), as described previously.^37^ Briefly, mice were trained to the apparatus for 1 week before baseline measurements. Following training, BP was assessed once a week for the duration of the experiment.

Cardiac function and structure were assessed by echocardiography using an Acuson Sequoia c512 ultrasound system to acquire noninvasive 2-dimensional–guided M-mode images at a 20 mm depth at the tip of the papillary muscles. For this procedure, mice were anesthetized with 1% isoflurane. Measurements were made in a short-axis view using the leading edge-to-lead edge convention during both systole and diastole over at least 3 consecutive cardiac cycles. Echocardiographic indices assessed included left ventricular anterior wall thickness and fractional shortening to assess left ventricular function. The ratio of peak velocity blood flow from ventricular relaxation in early diastole (the E wave) to peak velocity flow in late diastole (the A wave) was calculated from measuring blood velocities across the mitral valve during each cardiac cycle. Early (E) to late (A) ventricular filling velocities was calculated as an indirect measure of diastolic function. Ejection fraction=([left ventricular end-diastolic volume–left ventricular end-systolic volume]/left ventricular end-systolic diameter×100);fractional shortening=([left ventricular end-diastolic diameter–left ventricular end-systolic diameter]/left ventricular end-diastolic diameter×100).

### Assessment of Cardiac, Renal, and Splenic Size and Body Weight

Cardiac hypertrophy and splenic and renal size were assessed by measuring the organ weight to tibia length ratio. Body weight was measured weekly during the study.

### Quantification of Angiotensin and BK in Plasma and Kidney Samples

Blood and kidney samples were collected from mice under anesthesia with 5% isoflurane in 1.5 L/min O_2_. Cardiac puncture was used to collect blood from mouse. The quantification of endogenous levels of Ang requires a very rapid stabilization of the sample during collection. To ensure the complete inhibition of enzymes capable of converting angiotensin, blood was collected directly into a vial containing a proprietary mixture of ethylenediaminetetraacetic acid, pepstatin A, p-hydroxymercuribenzoic acid, phenanthroline, and other specific RAS inhibitors efficiently inactivating angiotensin metabolism (Attoquant Diagnostics). Samples were centrifuged for 10 minutes at 4 °C and 3000*g* to isolate plasma, which was maintained in −80 °C. Kidneys were snap-frozen in liquid nitrogen immediately following collection and stored at −80 °C. Vasoactive peptides were quantified in tissue samples using a procedure combining mechanical homogenization and detergent based extraction followed by liquid chromatography tandem mass spectrometry (LC-MS/MS) quantification (Attoquant Diagnostics). Briefly, frozen tissue was homogenized under liquid nitrogen using a pestle and mortar. The frozen tissue powder was dissolved in ice-cold 6 M aqueous guanidinium chloride, spiked with 200 pg of stable isotope-labeled internal standards for angiotensin peptide per milliliter of tissue extract. Standard spiked tissue extracts were subjected to a C18-based solid-phase extraction and analyzed by LC-MS/MS using a reversed-phase analytical column (CORTECS, UPLC C18, 1.6 μm; Waters) operating in line with a XEVO TQ-S triple quadrupole mass spectrometer (Waters) operated in multiple reaction monitoring mode. Two different mass transitions were measured per peptide, and the resulting concentrations were calculated provided that integrated signals for endogenous peptides exceeded a signal-to-noise ratio of 10.

### RAS Equilibrium Analysis and Assessment of BK Metabolism by LC-MS/MS

As proof of principle to evaluate whether the different drugs influence Ang and BK metabolism, RAS equilibrium analysis and analysis of BK metabolism were assessed in human plasma ex vivo as detailed below (Attoquant Diagnostics, Vienna).

Equilibrium concentrations of angiotensin metabolites were measured in human plasma by LC-MS/MS following ex vivo equilibration in the absence and presence of the indicated drugs, drug combinations, and recombinant enzymes. Plasma was collected from 6 healthy volunteers (3 males and 3 females, 35–48 years). Sacubitrilat, the active metabolite of sacubitril, was used in these ex vivo studies. rhACE2 (recombinant human ACE2) and rhNEP (recombinant human NEP) were added before equilibration to investigate the contribution of these predominantly endothelial enzymes to Ang equilibrium levels. Briefly, samples were spiked with stable isotope labeled internal standards after equilibration, and analytes were extracted using C18-based solid-phase extraction. Extracts of samples were analyzed using mass spectrometry analysis using a reversed-analytical column (Acquity UPLC C18, Waters) operating in line with a XEVO TQ-S triple quadrupole mass spectrometer (Waters Xevo TQ/S, Milford, MA) in multiple reaction monitoring mode. Internal standards were used to correct for analyte recovery across the sample preparation procedure in each individual sample. Analyte concentrations were calculated from integrated chromatograms considering the corresponding response factors determined in appropriate calibration curves in serum matrix, when integrated signals exceeded a signal-to-noise ratio of 10. The lower limits of quantification for equilibrium angiotensin levels in human plasma were 3 pmol/L (Ang I), 2 pmol/L (Ang 2–10), 1 pmol/L (Ang II), 2 pmol/L (Ang III), 1 pmol/L (Ang IV), 2 pmol/L (Ang 1–9), 2 pmol/L (Ang 1–7), 1 pmol/L (Ang 2–7), 1 pmol/L (Ang 3–7), and 1 pmol/L (Ang 1–5), respectively.

To assess BK metabolism, human plasma was supplemented with recombinant human ACE2 and NEP to investigate their role in plasma BK metabolism. Briefly, 10% human plasma supplemented with 200 pg/mL rhNEP or 200 pg/mL rhNEP and 1 µg/mL rhACE2 samples were spiked with BK 1 to 9 (50 ng/mL) in the presence and absence of the different drugs. Following C18-based solid-phase extraction, samples were subjected to LC-MS/MS analysis using a reversed-phase analytical column (Acquity UPLC C18, Waters) operating in line with a XEVO TQ-S triple quadrupole mass spectrometer (Waters Xevo TQ/S, Milford, MA) in multiple reaction monitoring mode. Internal standards were used to correct for analyte recovery across the sample preparation procedure in each individual sample. BK metabolite concentrations (BK 1–8, BK 1–7, BK 1–5, and BK 2–9) were calculated from integrated chromatograms considering the corresponding response factors determined in appropriate calibration curves in serum matrix, on condition that integrated signals exceeded a signal-to-noise ratio of 10.

### Myographic Assessment of Vascular Functional, Structural, and Mechanical Properties

Mesenteric arteries were studied because they are small vessels that play a role in peripheral resistance and hence in BP regulation. Perivascular tissue was removed, arteries were cut into 2 mm ring segments, and mounted on a wire myograph (Danish Myo Technology, Aarhus, Denmark) filled with 5 mL of physiological solution and continuously gassed with a mixture of 95% O_2_ and 5% CO_2_ while maintaining a temperature of 37 °C. The relationship between resting wall tension and internal circumference was determined, and the internal circumference, L100, corresponding to a transmural pressure of 100 mm Hg for a relaxed vessel in situ, was calculated. The vessels were set to the internal circumference L1, given by L1=0.9×L100. The effective internal lumen diameter was determined as L1=L1/π and was between 200 and 300 µm. After 60 minutes of stabilization, the contractile ability of the preparations was assessed by adding KCl solution (120 mmol/L) to the organ baths. The endothelium integrity was verified by relaxation induced by acetylcholine (10^-6^ mol/L) in mesenteric arteries precontracted with U46619 (10^-7^ mol/L). To check endothelium-dependent vasodilatation and endothelium-independent relaxation, acetylcholine, and sodium nitroprusside (SNP), respectively, were used in vessels precontracted with U46619 (10−7 mol/L) and vascular contraction was assessed by U46619.

Vascular structure and mechanical properties were assessed in resistance arteries prepared as pressurized systems on a pressure myograph, as previously described.^38^ For the assessment of structural and mechanical properties, second-order branches of the mesenteric artery (2–3 mm in length) were slipped onto 2 glass microcannula, one of which was positioned until vessel walls were parallel, in a pressure myograph (DMT myograph; ADInstruments Ltd, Oxford, United Kingdom). Vascular structure and mechanics were assessed under Ca^2+^-free conditions to eliminate the effects of myogenic tone. Vessels were perfused for 30 minutes with Ca^2+^ free Krebs solution containing 10 mmol/L EGTA. Measurement of media thickness and lumen diameter were taken at stepwise increments of luminal pressure (10–120 mm Hg). Vascular structural and mechanical parameters were calculated, as previously described.^38^

### In Vivo Assessment of Vascular Permeability by Evans Blue

Vascular permeabilization in response to NEP and ACE inhibitors was evaluated in vivo by Evans Blue assay, as described previously.^39,40^ Briefly, 200 µL sterile solution of 0.5% Evans Blue in PBS was intravenously injected through the tail vein in anesthetized mice. Animals were then sacrificed through cervical dislocation 30 minutes after administration. Organs were collected, weighed, and exposed to formamide for 24 hours at 55 °C. The mixture was centrifuged to pellet any remaining tissue fragments, and the absorbance at 610 nm was recorded by spectrophotometer using formamide as a blank. The microgram of Evans Blue extravasated per gram of tissue was calculated through the Evans Blue calibration curve and compared with control group.

### Macrophage Phenotype Assessed by Flow Cytometry

The left kidney from each mouse was isolated and transferred to PBS on ice. Kidneys were weighed, cut into small pieces, and digested in a mix of enzymes. Briefly, collagenase type XI (125 U/mL), collagenase type IS (450 U/mL), and hyaluronidase IV-S (60 U/mL) were used for digestion at 37 °C for 20 minutes, with regular agitation. The digested tissue was then passed through a 70 μm sterile cell strainer (Falcon; BD Biosciences, San Jose, CA) to yield a single-cell suspension. Cells were washed and resuspended in fluorescence-activated cell sorting buffer, counted, stained, and collected using multicolor flow cytometry (BD LSR II flow cytometer with DIVA software, BD Biosciences). The macrophage population was defined as CD45+F4/80+CD11b+ cells and further characterized for M1 proinflammatory macrophage (CD11c+) as previously described.^41^ Following antibodies were used in the panel: anti-CD45, anti-Ly-6G/Ly-6C, anti-CD11b, anti-CD11c (all from BioLegend), and anti-F4/80 (eBioscience). Dead cells were eliminated from analysis using Zombie Violet (BD Biosciences). For each experiment, fluorescence minus one control for each fluorophore was performed to establish gates. In selected experiments, accuracy of the fluorescence minus one gating strategy was confirmed using isotype controls. Data were analyzed by Flow Jo v.10 (Ashland, OR).

### Statistical Analysis

Data are expressed as mean±SEM unless otherwise stated. Statistical comparisons of parameters between groups were performed using a 2-tailed Student *t* test or 1-way and 2-way ANOVA followed by Dunnett or Bonferroni post hoc tests as appropriate. *P*<0.05 was considered statistically significant. Data analysis was conducted using GraphPad Prism 5.0.

## Results

### Effects of Treatment on Body Weight and Organ Size

Body weight increased gradually in both WT and LinA3 groups over the 4-week study period, with no differences between groups (Figure S2A). Treatment variably increased body weight in the normotensive and hypertensive mice (Figure S2A through S2G). LisW-S with or without sacubitril increased body weight in LinA3 and WT mice, whereas lisinopril increased body weight in LinA3 mice (Figure S2C and S2D). Sacubitril alone and omapatrilat effects on body weight were minimal (Figure S2B and S2G).

Heart weight was significantly increased in LinA3 mice versus controls (Figure S3A). Kidney and splenic weight were not altered in LinA3 mice (Figures S3B and S3C). All treatments prevented development of cardiac hypertrophy in hypertensive mice (Figure S3D through S3I).

Despite no change in kidney size, there was significant renal inflammation in LinA3 mice, as evidenced by a significant increase in frequency of M1 macrophages (Figure S4), which was reduced by treatment with all drugs (Figure S4B through S4G).

### Combined ACE C-Domain Inhibitor (LisW-S) and NEP Inhibitor (Sacubitril) Treatment Reduces BP and Improves Cardiac Function

**Figure 1.**
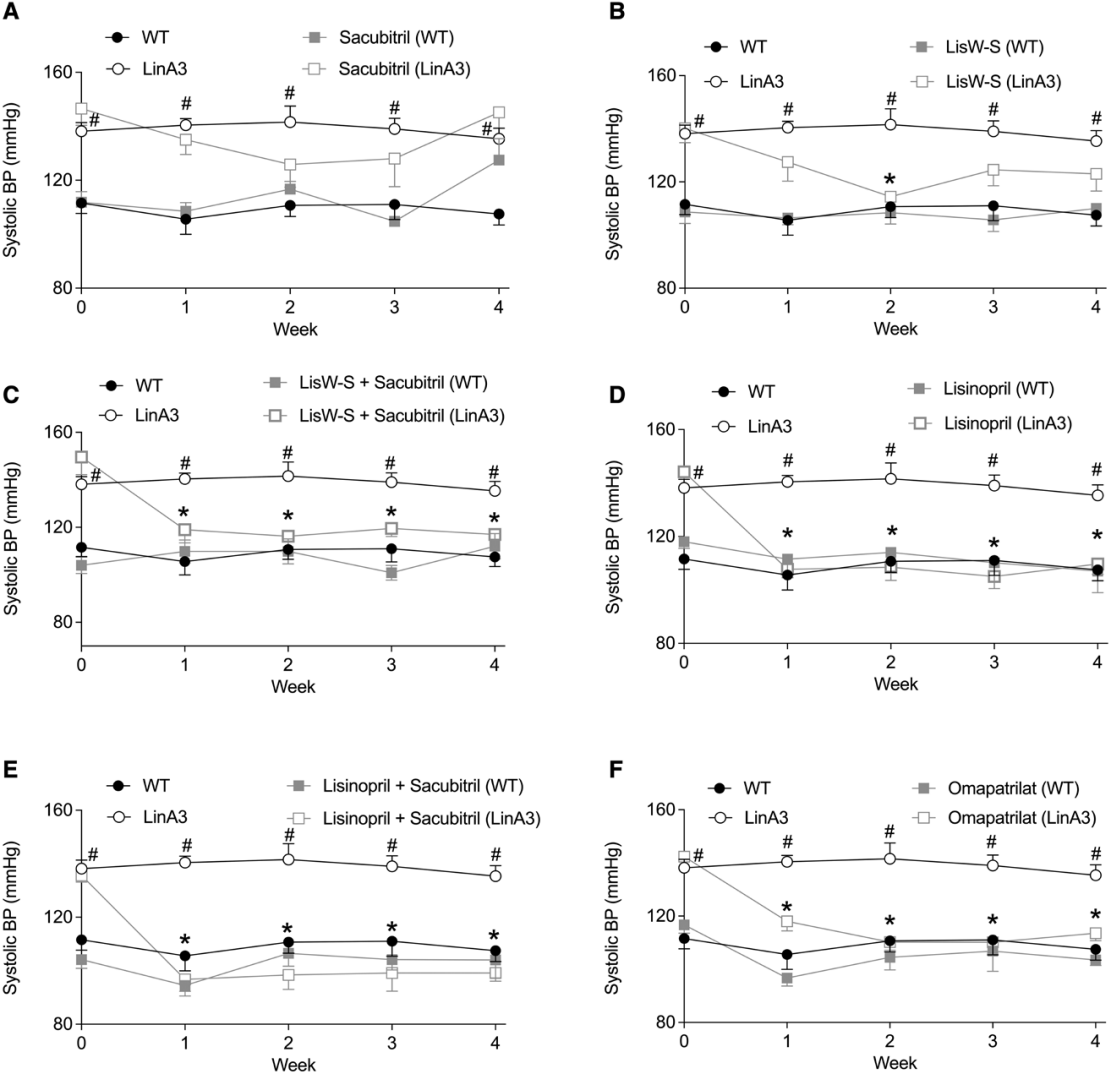
**Effects of treatment on systolic blood pressure (BP).** Wild-type (WT) and LinA3 mice were treated with sacubitril (**A**), lisinopril-tryptophan (lisW-S; **B**), lisW-S+sacubitril (**C**), lisinopril (**D**), lisinopril+sacubitril (**E**), and omapatrilat (**F**). Blood pressure was measured by tail cuff for 4 wk and 1 wk before starting the treatment. Data are mean±SEM (n=6; 2-way ANOVA with Bonferroni post hoc test). *P*<0.05. *vs LinA3; #vs WT.

Systolic BP was significantly increased in LinA3 mice (Figure S12A). The increase in BP was sustained during the experimental period. Sacubitril induced a transient, nonsignificant reduction in BP in LinA3 mice (Figure 1A). ACE C-domain inhibitor lisW-S reduced BP after 2 weeks of treatment (Figure 1B). The combination lisW-S+sacubitril reduced systolic BP after 1 week of treatment, which was sustained during the experimental period (Figure 1C). The sustained BP-lowering effects after 1 week of treatment in lisW-S+sacubitril mice were similar to BP effects in mice treated with lisinopril, lisinopril+sacubitril, and omapatrilat (Figures 1D through 1G).

To evaluate cardiac function, echocardiography was performed at the end of the treatment period. LinA3 mice exhibited reduced fractional shortening (Figure S5A), an effect that was ameliorated by treatment with lisW-S, lisW-S+sacubitril, lisinopril, and omapatrilat (Figure S5B through S5G). Anterior wall thickness (Figure S6) and the ratio of early to late ventricular filling velocities (Figure S7) were not significantly different between WT and LinA3 mice in basal and treatment conditions.

Sacubitril combined with lisW-S reverses vascular hypercontractility and endothelium-independent vasorelaxation without affecting endothelium-dependent vasorelaxation.

**Figure 2.**
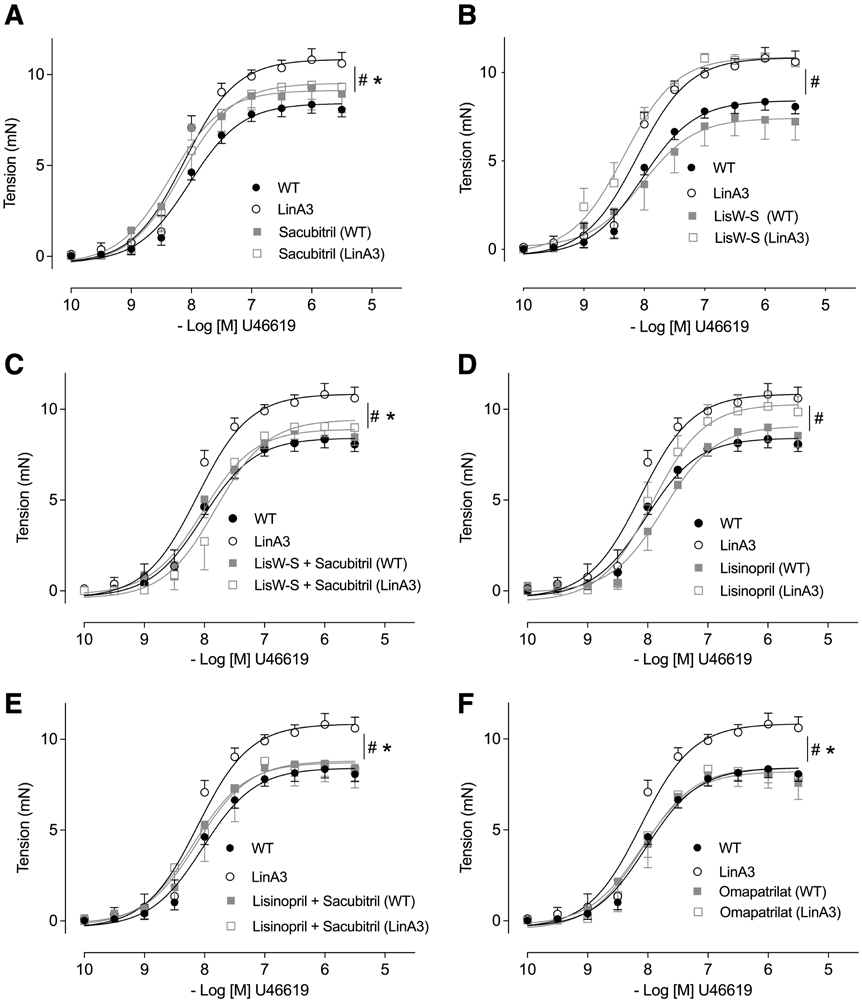
**Effects of treatment on vascular contraction.** Concentration-response curves to U46619 were performed in mesenteric arteries isolated from wild-type (WT) and LinA3 mice treated with sacubitril (**A**), lisinopril-tryptophan (lisW-S; **B**), lisW-S+sacubitril (**C**), lisinopril (**D**), lisinopril+sacubitril (**E**), and omapatrilat (**F**) and mounted on wire myograph. U46619 tension curves (contraction) were expressed in mN and represent the mean±SEM (n=5–6; 2-way ANOVA with Bonferroni post-test). *P*<0.05. *vs WT. #vs LinA3.

To evaluate the vascular effects of treatment in hypertensive mice, mesenteric resistance arteries isolated from WT and LinA3 mice were mounted on wire myographs and vascular function was evaluated in untreated- and drug-treated mice. The vasoconstrictor U46619 increased contraction in a dose-dependent manner in arteries from WT and LinA3 mice, with significantly increased contractile response in hypertensive mice (Figure S12B). Increased contraction in LinA3 mice was prevented by sacubitril alone or combined with lisW-S or lisinopril but was unaffected by lisW-S and lisinopril alone (Figure 2A through 2E). Omapatrilat normalized contractile response in hypertensive mice (Figure 2F).

**Figure 3.**
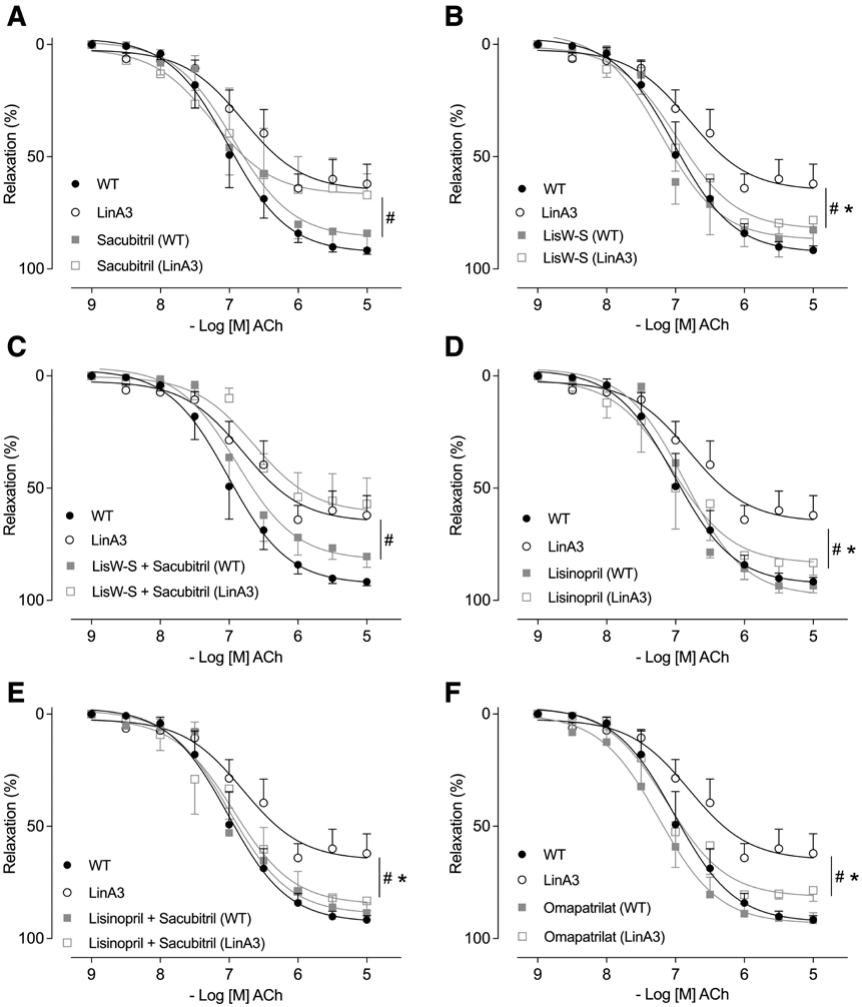
**Inhibition of ACE (angiotensin-converting enzyme) C-domain (lisinopril-tryptophan [lisW-S]) combined with NEP (neprilysin) inhibitor (sacubitril) does not reverse impaired endothelial-dependent relaxation in hypertensive mice.** Concentration-response curves to acetylcholine (Ach) were performed in mesenteric arteries isolated from wild-type (WT) and LinA3 mice treated with sacubitril (**A**), lisW-S (**B**), lisW-S+sacubitril (**C**), lisinopril (**D**), lisinopril+sacubitril (**E**), and omapatrilat (**F**) and mounted on wire myograph. Ach curves (relaxation) were expressed in % of relaxation compared with preconstriction induced by U44619 and represented as mean±SEM (n=5–6; 2-way ANOVA with Bonferroni post-test). *P*<0.05. *vs WT. #vs LinA3.

Endothelium-dependent relaxation was assessed by evaluating acetylcholine-induced vasodilation in preconstricted vessels from untreated- and treated-WT and LinA3 mice (Figures S12C, S3A through S3F). Mesenteric arteries from LinA3 mice exhibited impaired acetylcholine-induced vascular relaxation, as evidenced by significantly reduced maximum responses to acetylcholine (Figure S12C). Although sacubitril alone and lisW-S+sacubitril did not influence Ach-induced vasorelaxation in LinA3 mice (Figure 3A and 3C), lisW-S, lisinopril, lisinopril+sacubitril, and omapatrilat normalized responses (Figure 3B, 3D through 3F).

Endothelium-independent vasorelaxation was assessed in arteries exposed to SNP. As shown in Figure S8, SNP-induced vasorelaxation was significantly reduced in LinA3 mice. All treatments improved endothelium-independent vasorelaxation in hypertensive mice as evidenced by normalization of SNP-induced vasorelaxation in the LinA3 group (Figure S8B through S8G).

### Inhibition of NEP and ACE Ameliorates Vascular Remodeling and Impaired Mechanical Properties in LinA3 Mice

In addition to vascular function, we assessed effects of the different treatments on vascular structure and mechanical properties. Mesenteric arteries were mounted on myographs as pressurized systems. Hypertensive mice exhibited significant remodeling, with the wall/lumen ratio, cross-sectional area, and wall thickness significantly increased in LinA3 mice compared with WT mice (Figures S13, S9, and S10). These parameters were normalized by the different treatments (Figures S13B and S13F, S9B through 9SG, and S10B through S10G). Vascular elasticity was also measured in mesenteric arteries from WT and hypertensive mice. Vascular elasticity was reduced in LinA3 mice (Figure S14), as indicated by a leftward shift in the stress:strain curve. This effect was ameliorated only by lisinopril+sacubitril and omapatrilat. The other treatments had no effect on vascular mechanical properties in hypertensive mice.

### Inhibition of NEP Combined With ACE C-Domain Reduces Ang II Production and Increases BK Metabolism

**Figure 4.**
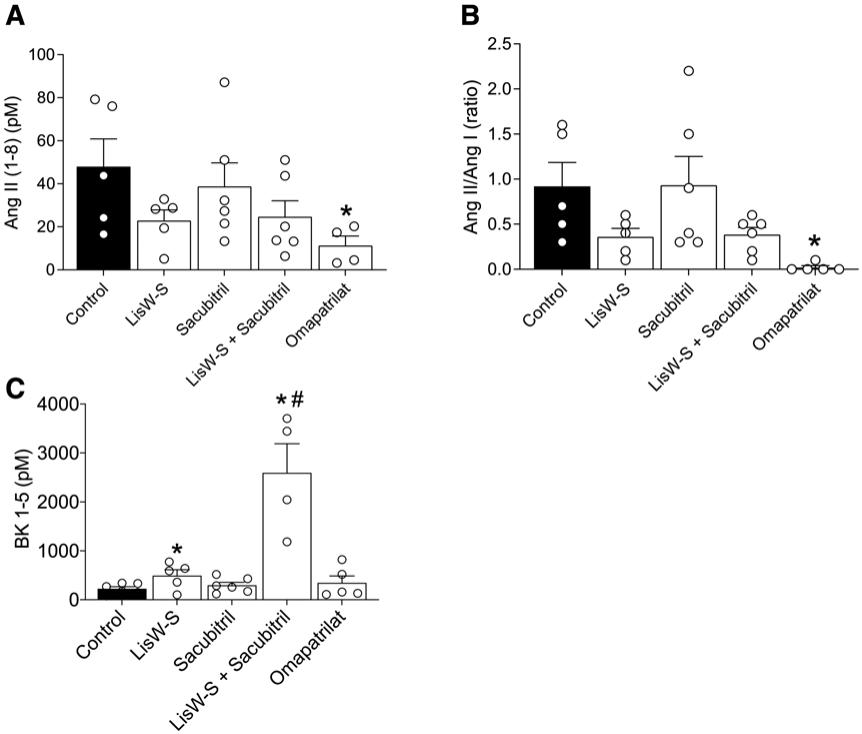
**Effects of treatments on plasma angiotensin and BK (bradykinin) levels.** Ang II (angiotensin II; **A**) and Ang II/Ang I (**B**) peptides were quantified in plasma isolated from LinA3 mice by liquid chromatography tandem mass spectrometry (LC-MS/MS) quantification. Peptides were measured in samples isolated from control, lisinopril-tryptophan (lisW-S), sacubitril alone or combined with lisW-S, and omapatrilat-treated mice. Plasma BK 1–5 levels (**C**) were measured in LinA3 mice by LC-MS/MS. Data represent the means±SEM (n=4–9; 1-way ANOVA with Dunnett post-test). *P*<0.05 vs control (*) or vs lisW-S (#).

To evaluate if improvement of vascular function in animals treated with lisW-S+sacubitril was associated with changes in Ang and BK levels, we profiled these peptides in plasma and tissues isolated from WT and LinA3 mice. Ang I levels in kidney (Figure S11A) were not changed by treatments. Ang II levels were reduced in kidneys isolated from LinA3 mice treated with lisW-S alone or combined with sacubitril and mice treated with omapatrilat (Figure S11B and S11C). Plasma Ang II and Ang II/Ang I levels were reduced in animals treated with omapatrilat (Figure 4A and 4B).

Levels of the stable BK metabolite, BK 1 to 5 (which has no known functional activity), were assessed in plasma from LinA3 mice. As shown in Figure 4C, BK 1 to 5 levels were significantly increased in LinA3 mice treated with lisW-S. This response was further increased when mice were treated with lisW-S+sacubitril. Plasma BK 1 to 5 levels were not altered in mice treated with lisinopril, sacubitril, or omapatrilat.

**Figure 5.**
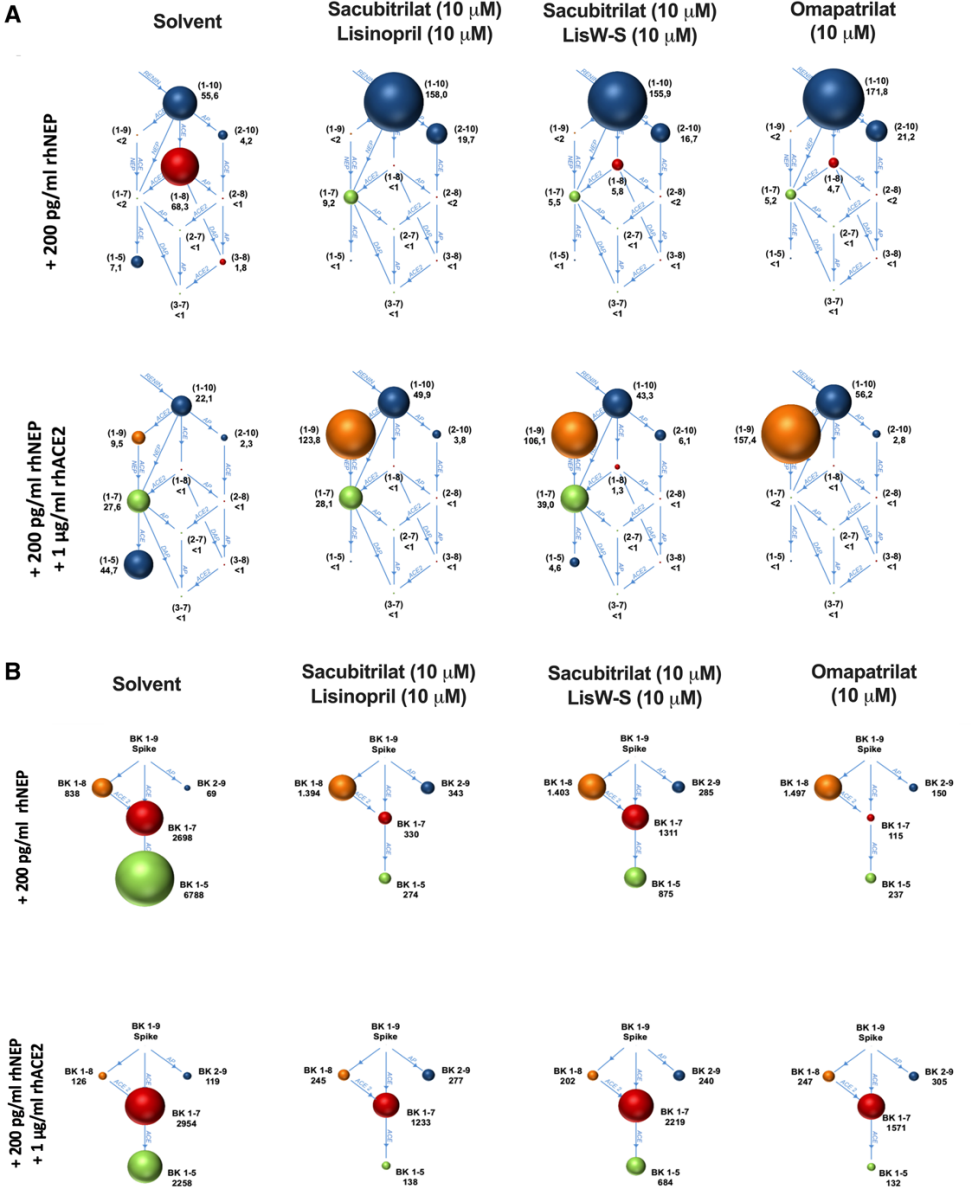
**Effect of ACE (Ang [angiotensin]-converting enzyme) and NEP (neprilysin) inhibitors on angiotensin and BK (bradykinin) metabolite levels in equilibrated blood plasma.A**, Mean blood plasma equilibrium concentrations of Ang metabolites in human plasma supplemented with rhNEP (recombinant human NEP; 200 pg/mL) and rhACE2 (recombinant ACE2; 1 µg/mL) on top of rhNEP (200 pg/L) in the presence of the indicated compounds are shown. Ang metabolites are represented by spheres where the diameters reflect the concentration of the peptide. The metabolite sequence is given in brackets and the peptide concentration is given in pmol/L next to each sphere. Enzymes known to convert one peptide to another are indicated in blue. Ang I (Ang 1–10) (top blue sphere) is converted to the potent vasoconstrictor peptide AngII (Ang 1–8) (red sphere) by ACE. ACE2 is responsible for the conversion of Ang I to Ang (1–9) (orange sphere) and the conversion of Ang II to cardioprotective peptide Ang 1–7 (green sphere). Ang 1–7 is also formed by ACE and NEP-mediated cleavage of Ang 1–9 and is degraded by ACE, AP (aminopeptidases), and DAP (dipeptidyl aminopeptidase). **B**, BK metabolites in human plasma exposed to the different drugs. Human plasma (10%) supplemented with 200 pg/mL rhNEP (**A**) or 200 pg/mL rhNEP and 1 µg/mL rhACE2 (**B**) was spiked with BK (1–9) (50 ng/mL) in the presence and absence of the indicated compounds. BK metabolites are represented by spheres where the diameters reflect the concentrations of the respective peptides. The metabolite sequence is given in brackets and the concentration is given in pmol/L next to each sphere. Enzymes known to convert one peptide to another are indicated in blue.

As proof of concept related to differential drug effects on Ang and BK metabolites, we also performed ex vivo studies in human plasma (Figure 5). Figure 5A shows the RAS equilibrium fingerprint, which demonstrates a similar hydrolysis of Ang 1 to 10 by lisW-S+sacubitrilat and omapatrilat. Figure 5B shows that the hydrolysis of BK 1 to 9 in human plasma is greater with lisW-S+sacubitrilat than with the other treatments. This is indicated by the larger diameters of the spheres (representing BK 1–7 and BK 1–5 concentrations) in the lisW-S+sacubitrilat group compared with the other groups.

### Omapatrilat but Not lisW-S+Sacubitril Increases Vascular Permeability

**Figure 6.**
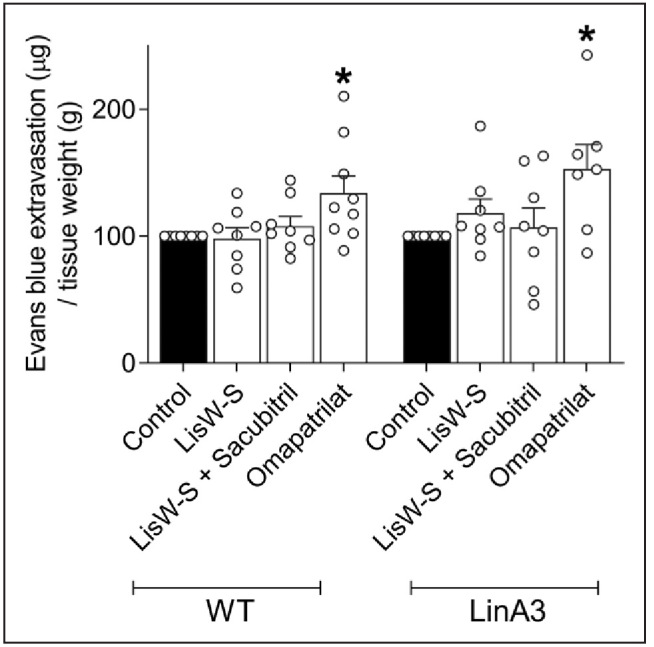
**Omapatrilat but not lisinopril-tryptophan (lisW-S)+sacubitril increases vascular permeability.** Vascular permeability was measured by Evans Blue extravasation. Values express Evans Blue extravasation normalized by tissue weight±SEM (n=7–9; 1-way ANOVA with Dunnett post-test). *P*<0.05. *vs wild-type (WT) control; #vs LinA3 control.

We used the Evans Blue assay to assess vascular permeability in WT and LinA3 mice treated with lisW-S, lisW-S+sacubitril, or omapatrilat. Normally, the intact endothelium is impermeable to albumin, and accordingly, Evans Blue bound to albumin remains within blood vessels. With vascular injury, permeability increases, and the endothelium becomes permeable to albumin, causing extravasation of Evans Blue in tissue.^39,40^ As shown in Figure 6, Evans Blue extravasation was significantly increased by omapatrilat in WT and LinA3 mice, an effect that was not evident in mice treated with lisW-S or lisW-S+sacubitril.

## Discussion

Despite the availability of many classes of antihypertensive drugs, control of hypertension remains suboptimal in the majority of treated patients, highlighting the need for new therapeutic strategies.^42,43^ To address this, we investigated the BP and cardiovascular properties of a novel drug combination, C-domain–selective ACE inhibitor (lisW-S) combined with a NEP inhibitor (sacubitril), in a mouse model of Ang II–dependent hypertension, and compared the effects to those of omapatrilat, which inhibits ACE C-domain and N-domain and NEP (recapitulated by lisinopril+sacubitril in our study). Major findings in our study demonstrate that (1) lisW-S combined with sacubitril reduced systolic BP in LinA3 mice similar to that of omapatrilat, (2) unlike omapatrilat, lisW-S+sacubitril did not modify endothelium-dependent vasorelaxation, (3) omapatrilat but not lisW-S+sacubitril caused plasma extravasation and vascular leakage, and (4) lisW-S+sacubitril, but not omapatrilat, increased plasma levels of BK 1 to 5. These findings suggest that targeting the C-domain of ACE while preserving the activity of the ACE N-domain combined with NEP inhibition may be as effective as omapatrilat in lowering BP and cardiovascular protection, but without injurious effects on endothelial permeability, which is implicated clinically in angioedema.

Activation of C-domain ACE is the predominant source of Ang II and is critically involved in BP elevation.^29,30^ We previously demonstrated that, unlike lisinopril, lisW-S significantly decreased BP without the accumulation of BK, implying that selective targeting of the C-domain of ACE may minimize the potential injurious effects of BK.^29,30^ Here, we have advanced this paradigm by exploring the putative added benefits of simultaneously inhibiting NEP and the ACE C-domain. Combination lisW-S+sacubitril, lisinopril+sacubitril, and omapatrilat were all equally effective in reducing BP and preventing hypertension-associated cardiac dysfunction (hypertrophy and systolic dysfunction) in hypertensive mice. Importantly, combination of lisW-S plus sacubitril reduced Ang II production while regulating BK levels, due to NEP inhibition, likely leading to a sustained BP lowering in LinA3 mice. However, lisW-S alone was ineffective in maintaining BP reduction, which corroborates previous publications indicating a putative role for BK in antihypertensive effects of ACE inhibitors.^44^

Sacubitril alone was ineffective in sustained BP reduction and had no effect on cardiac hypertrophy or left ventricular fractional shortening and systolic function, similar to what has been previously demonstrated in experimental and clinical hypertension.^12^ This has been attributed to the influence of NEP on the metabolism of the vasoconstrictors Ang II and endothelin-1, and Ang-(1–7) production from Ang I. NEP inhibition, in addition to influencing BK and natriuretic peptides, causes accumulation of vasopressors Ang II and endothelin-1, and reduces production of Ang-(1–7), which is vasoprotective.^12^ This may explain, at least partially, why chronic use of sacubitril is not an effective antihypertensive agent.

To explore potential vascular mechanisms involved in BP reduction by the various treatments, we assessed the functional, structural, and mechanical properties of resistance arteries. Hypertensive mice exhibited increased vascular contraction and impaired endothelium-dependent and -independent vasorelaxation. This was associated with structural and mechanical changes including increased wall:lumen ratio, wall thickness, and cross-sectional area, which are characteristic of hypertrophic remodeling, and reduced elasticity. These hypertension-associated vascular changes were variably improved by the different treatments and closely associated with a reduction in BP. Omapatrilat and lisinopril+sacubitril had similar effects on vascular contraction, endothelial dysfunction, and arterial remodeling in hypertensive mice, and both treatments reduced tissue Ang II levels, again demonstrating the efficacy of combination ACE and NEP inhibition. However, although lisW-S+sacubitril improved vascular hyperreactivity and endothelium-independent vasorelaxation (SNP-induced response) in LinA3 mice, this drug combination did not improve endothelium-dependent vasorelaxation. The reasons for this are unclear, but it is possible that mechanisms involved in endothelial relaxation, possibly nitric oxide, and EDRF production, may be impaired when the ACE N-domain is active while ACE C-domain and NEP are inhibited. The exact processes underlying this require further clarification. Nevertheless, the combination of improved vascular contraction and endothelium-independent vasorelaxation together with no change in endothelium-dependent vasodilation following lisW-S+sacubitril treatment suggests that this drug combination primarily targets vascular smooth muscle cells in the vascular media rather than endothelial cells in the endothelium.

Our findings related to vascular permeability substantiate the notion that lisW-S+sacubitril may be more vascular-protective than endothelial-protective. Despite similar BP-lowering effects of lisW-S+sacubitril and omapatrilat, these drugs had variable effects on the endothelium. In particular, endothelium-dependent vasorelaxation and vascular permeability were not altered by lisW-S+sacubitril, whereas endothelial injury in hypertensive mice was worsened by omapatrilat as shown in the Evans Blue studies. These findings support previous studies where omapatrilat increased fluid and protein permeability in cat skeletal muscle through BK-dependent processes^45^ and corroborate clinical data where omapatrilat caused unexpected tissue swelling and angioedema.^22,23^ Putative underlying processes have been attributed to inhibition of the breakdown of BK and substance P by ACE and aminopeptidases.^13,46^ Here, we observed that mice treated with LisW-S+sacubitril, but not omapatrilat, had increased BK 1-5 levels, indicating effective BK metabolism. This is in line with findings observed in our ex vivo studies demonstrating that LisW-S+sacubitril but not omapatrilat induces breakdown of BK to generate BK 1 to 5. Angioedema is thought to be mediated, at least in part, by BK accumulation and decreased BK metabolism.^47^ In our study, omapatrilat-treated mice did not exhibit significant BK breakdown, unlike the LisW-S+sacubitril–treated mice, reinforcing the concept that preserving ACE N-domain activity in the presence of NEP inhibition promotes BK metabolism, which may be linked to decreased BK accumulation and vascular permeability and possibly reduced angioedema risk.

To explore effects of treatment on vascular permeability, we examined plasma extravasation using the well-established Evans Blue method. Omapatrilat-treated mice exhibited a significant increase in Evans Blue staining, indicative of increased endothelial permeability. This phenomenon was absent in mice treated with lisW-S+sacubitril where ACE N-domain activity was preserved. These observations suggest that reduced ACE N-domain activity, rather than NEP, may be an important mechanism underlying BK accumulation and endothelial permeability. Other studies have shown that *NEP* knockout mice and mice treated with NEP inhibitors thiorphan and phosphoramidon increased vascular permeability via BK and neurokinin-1 receptor-mediated signaling.^13,47–49^ A major challenge for therapies targeting ACE and NEP is to achieve the beneficial cardiovascular effects without adverse consequences. Here, we present a new strategy that selectively blocks Ang I conversion to Ang II combined with NEP inhibition, which leads to a reduction in BP in a profile similar to that of omapatrilat, but without affecting endothelial function, vascular permeability, and BK degradation.

Increasing evidence indicates that hypertension is a proinflammatory disorder characterized by increased levels of inflammatory markers.^50,51^ Here, we corroborate this, as the abundance of proinflammatory M1 macrophages was increased in the kidneys of hypertensive mice. Activation of ACE N-domain is associated with inflammation.^52^ Our findings support this notion since lisW-S alone did not influence the inflammatory response (macrophage count) in LinA3 mice but was reduced when sacubitril was added, similar to effects of omapatrilat. These responses were independent of changes in kidney or spleen size, and therefore are likely to reflect local renal inflammatory events. Benefits of treatment may be due to direct anti-inflammatory actions of ACE+NEP inhibition but may also be secondary effects associated with a reduction in BP in the hypertensive mice.

In conclusion, the combination of a C-domain selective ACE inhibitor plus a NEP inhibitor in hypertensive mice reduces BP and normalizes vascular contraction without influencing endothelial function and vascular permeability. These features may be beneficial in the treatment of hypertension but without the possible side effects of angioedema associated with the inhibition of NEP and the ACE C- and N-domains. This has important clinical relevance and may be a potentially new strategy in the treatment of hypertension and associated cardiovascular diseases.

## Perspectives

Targeting the ACE C-domain, while preserving the ACE N-domain, combined with NEP inhibition may be as effective as omapatrilat in lowering BP and cardiovascular protection but without injurious endothelial effects that predispose to microvascular leakage implicated in angioedema. We have identified a clinically relevant strategy to inhibit ACE while simultaneously amplifying the natriuretic peptides system with minimal undesired effects, such as microvascular leakage and tissue edema, which are associated with BK accumulation.

## Sources of Funding

The authors are funded by grants from the British Heart Foundation (BHF) (RE/13/5/30177; 18/6/34217), the Medical Research Council (MC-PC-15076), and the Mobility Plus (1300/1/MOB/IV/2015/0). R.M. Touyz is supported through a BHF Chair award (CH/12/29762). A.C. Montezano is supported through a University of Glasgow Walton Fellowship in Cardiovascular Medicine.

## Acknowledgments

We thank Wendy Beattie for her help with our mice colonies, Laura Haddow and John McAbeny from the British Heart Foundation (BHF) Myography & Imaging Core Facility and Jackie Thomson and Ross Hepburn for laboratory support. Dr Mario Ehlers is thanked for expert input.

## Disclosures

E. Sturrock is a Director of AngioDesign (UK) Ltd. The other authors report no conflicts.

## Supplementary Material



## Novelty and Significance

### What Is New?

- We assessed the antihypertensive and cardiovascular effects of a novel drug strategy, combination of NEP (neprilysin) inhibitor and C-domain specific ACE (angiotensin-converting enzyme) inhibitors, and showed that unlike inhibitors that target NEP and the ACE N- and C-domains, beneficial effects are not associated with BK (bradykinin) accumulation or microvascular leakage.

### What Is Relevant?

- Combined ACE C-domain inhibitor (lisinopril-tryptophan) and NEP inhibitor (sacubitril) treatment reduced blood pressure and improved cardiac function.
- Sacubitril combined with lisinopril-tryptophan reversed vascular hypercontractility and endothelium-independent vasorelaxation, without affecting endothelium-dependent vasorelaxation.
- Combination inhibition of NEP and the ACE C-domain, but not inhibition of NEP+ACE N- and C-domains (omapatrilat), increased plasma BK 1-5 levels, possibly indicating BK metabolism.
- Vascular permeability was increased by omapatrilat but not by lisinopril-tryptophan+sacubitril.

### Summary

Lisinopril-tryptophan combined with a NEP inhibitor may have the same antihypertensive and cardiovascular favorable effects as omapatrilat, but without the undesirable properties possibly attributed to decreased BK breakdown.
