# Supplementary material for: Selective Inhibition of the C-Domain of ACE (Angiotensin-Converting Enzyme) Combined With Inhibition of NEP (Neprilysin): A Potential New Therapy for Hypertension
Source: Hypertension. 2021 Jul 26;78(3):604–16. doi: 10.1161/HYPERTENSIONAHA.121.17041 (PMC8357049; doi:10.1161/HYPERTENSIONAHA.121.17041)
Supplement: Supplementary file 1 [file hyp-78-604-s001.pdf]

DATA SUPPLEMENT

Selective inhibition of the C-domain of angiotensin converting enzyme combined with inhibition of neprilysin: A potential new therapy for hypertension

*Rhéure Alves-Lopes, PhD[1]; Augusto C Montezano, PhD[1]; Karla B Neves, PhD[1]; Harvey, Adam, PhD[1]; Francisco J Rios, PhD[1]; Dominik S Skiba, PhD[1]; Lauren B Arendse, PhD[3]; Tomasz J Guzik, MD, PhD[1]; Delyth Graham, PhD [1]; Marko Poglitsch, PhD [2]; Edward Sturrock, PhD [3]; *Rhian M Touyz, MD, PhD[1]

[1]Institute of Cardiovascular and Medical Sciences, University of Glasgow, UK; [2]Attoquant Diagnostics GmbH, Vienna, Austria; [3] Institute of Infectious Disease and Molecular Medicine and Division of Medical Biochemistry, University of Cape Town, South Africa.

***Corresponding authors:**
Rhéure Alves-Lopes, PhD and Rhian M Touyz, MD, PhD
Institute of Cardiovascular and Medical Sciences
University of Glasgow
126 University Place
Glasgow G12 8TA
Email address: Rheure.Lopes@glasgow.ac.uk; Rhian.Touyz@glasgow.ac.uk
Telephone number: 014 1330-7775

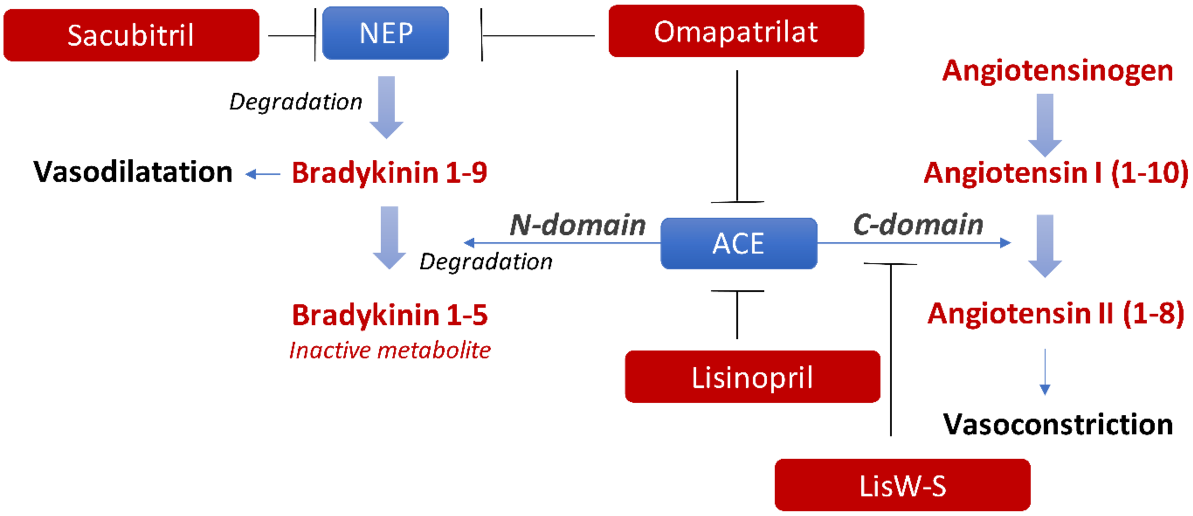

Figure S1. Mechanism of action of each drug used in this study. Omapatrilat is a drug that inhibits neutral endopeptidase and both domains of angiotensin converting enzyme. Lisinopril inhibits both domains of angiotensin converting enzyme. Sacubitril is a NEP inhibitor and lisW-S is an ACE C-domain-selective inhibitor derivative of lisinopril.

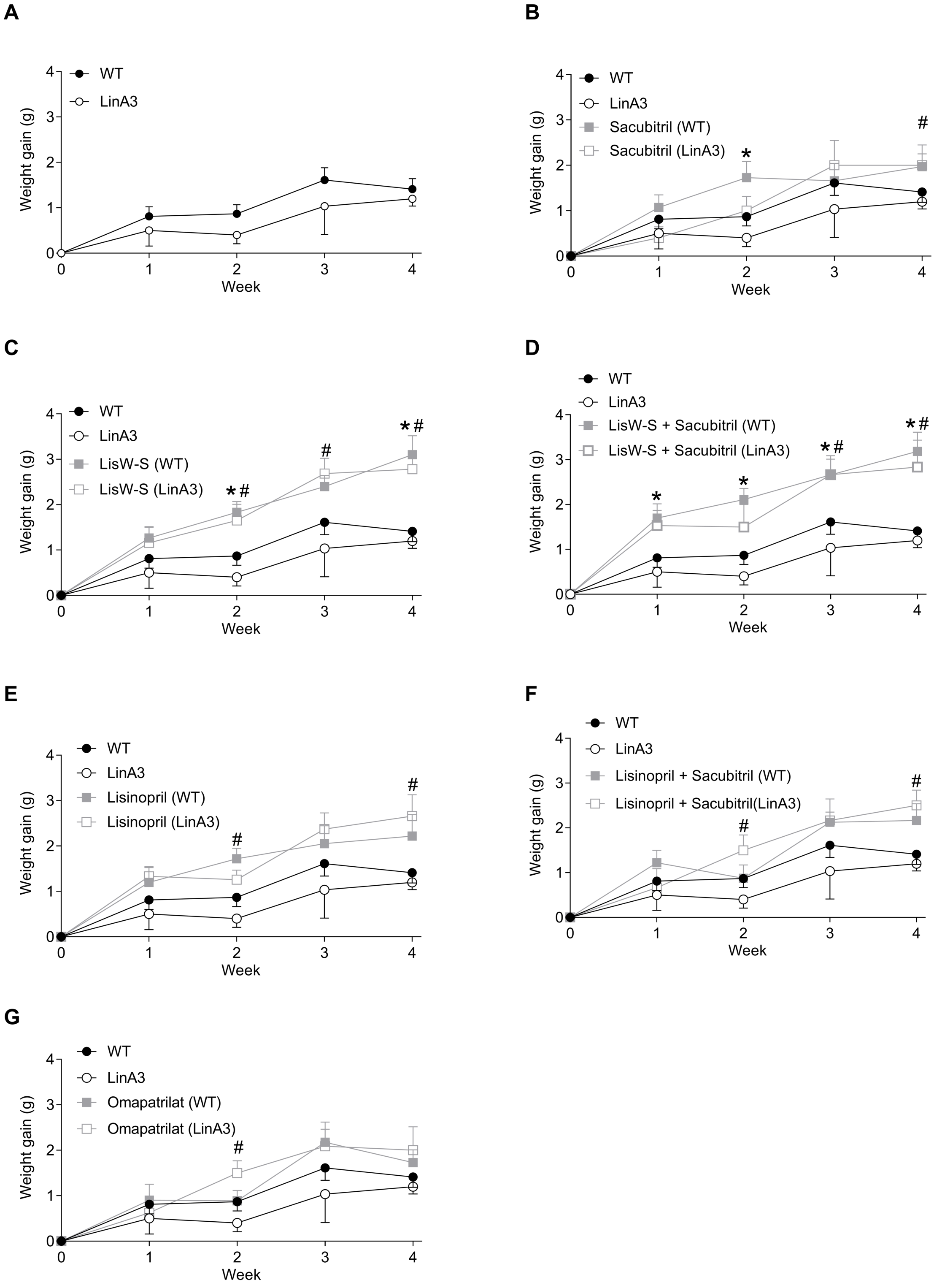

Figure S2. Inhibition of NEP and ACE system increases weight gain in normotensive and hypertensive mice. Weight gain was assessed for 5 weeks in WT and LinA3 mice controls (A) and treated with sacubitril (B), lisW-S (C), lisW-S + sacubitril (D), lisinopril (E), lisinopril + sacubitril (F) and omapatrilat (G). Values express gain weight and represent the mean±SEM (n=5-9; two-way ANOVA with Bonferroni's post hoc test). P<0.05. *: *vs* WT control; $^{\#}$: *vs* LinA3 Control.

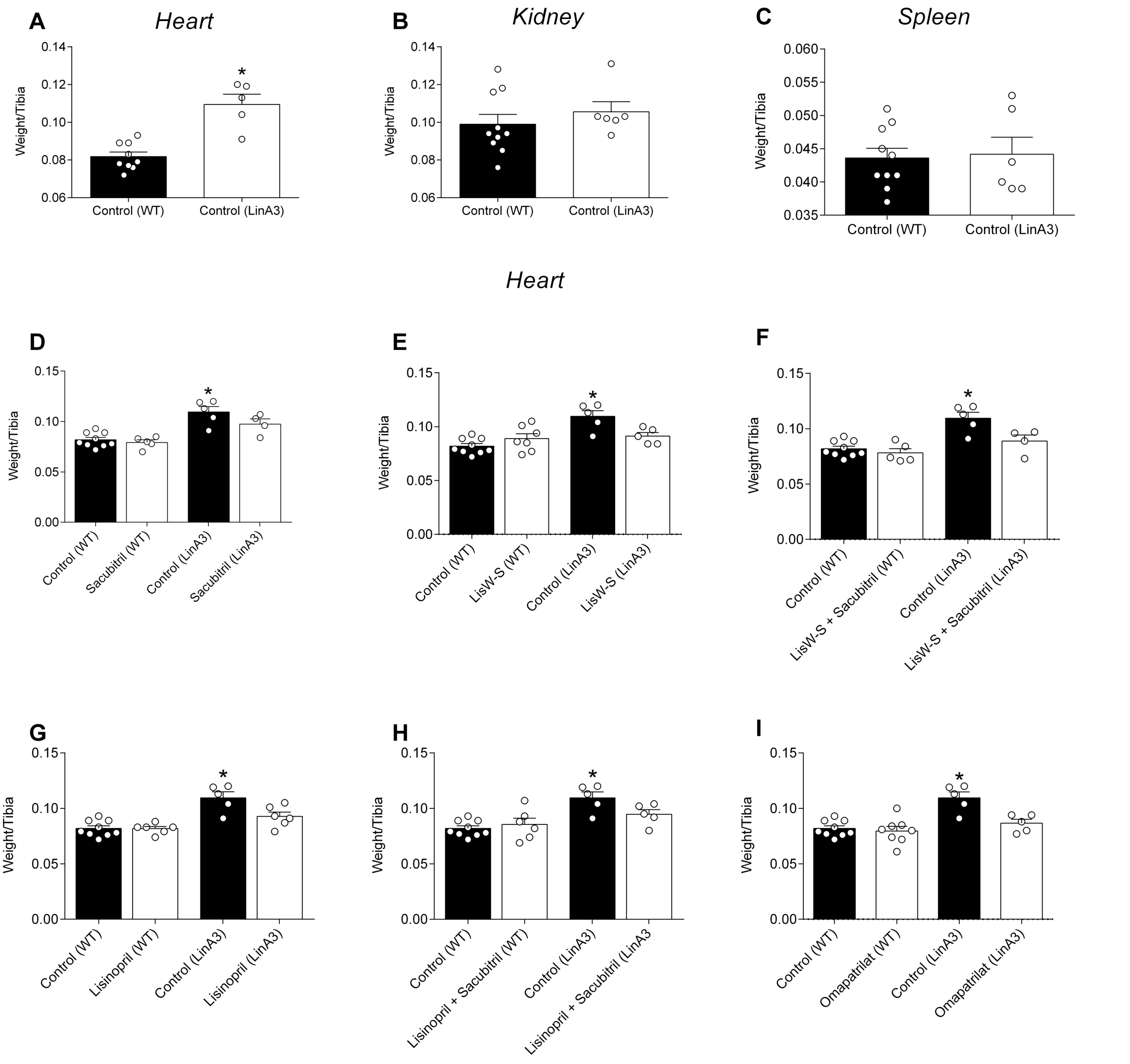

Figure S3. Inhibition of NEP and ACE system prevents increase in heart weight observed in LinA3 mice. Tissue weight of heart (A, D-I), kidney (B) and spleen (C) was measured after 4 weeks of treatment with sacubitril (D), lisW-S (E), lisW-S + sacubitril (F), lisinopril (G), lisinopril + sacubitril (H) and omapatrilat (I). Values express tissue weight and represent the mean±SEM (n=5-9; One-way ANOVA with Dunnett post-test). $P<0.05$. *: *vs* WT control.

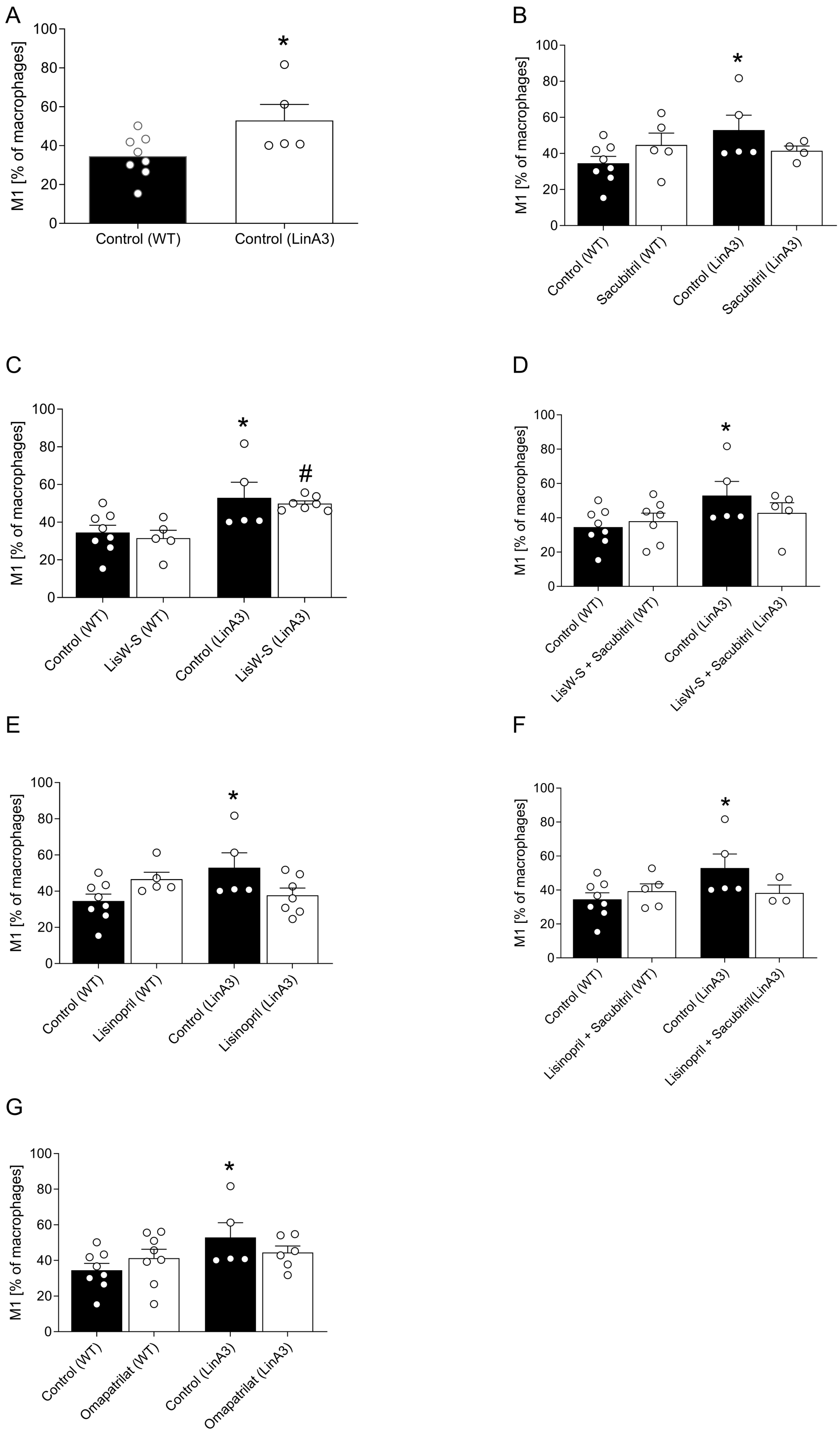

Figure S4. Increased M1 macrophages phenotype in kidneys from hypertensive mice is not observed in animal treated with ACE and NEP inhibitors. Macrophages phenotype was measured by flow cytometry (BD LSR II with DIVA software - BD Biosciences). Kidneys were isolated from WT and LinA3 mice controls (A) or treated with sacubitril (B), lisW-S (C), lisW-S + sacubitril (D), lisinopril (E), lisinopril + sacubitril (F) and Omapatrilat (G). Values were expressed in % of macrophages ± SEM (n=3-8; One-way ANOVA with Dunnett post-test). P<0.05. * *vs* WT. [#]: *vs* WT (LisW-S).

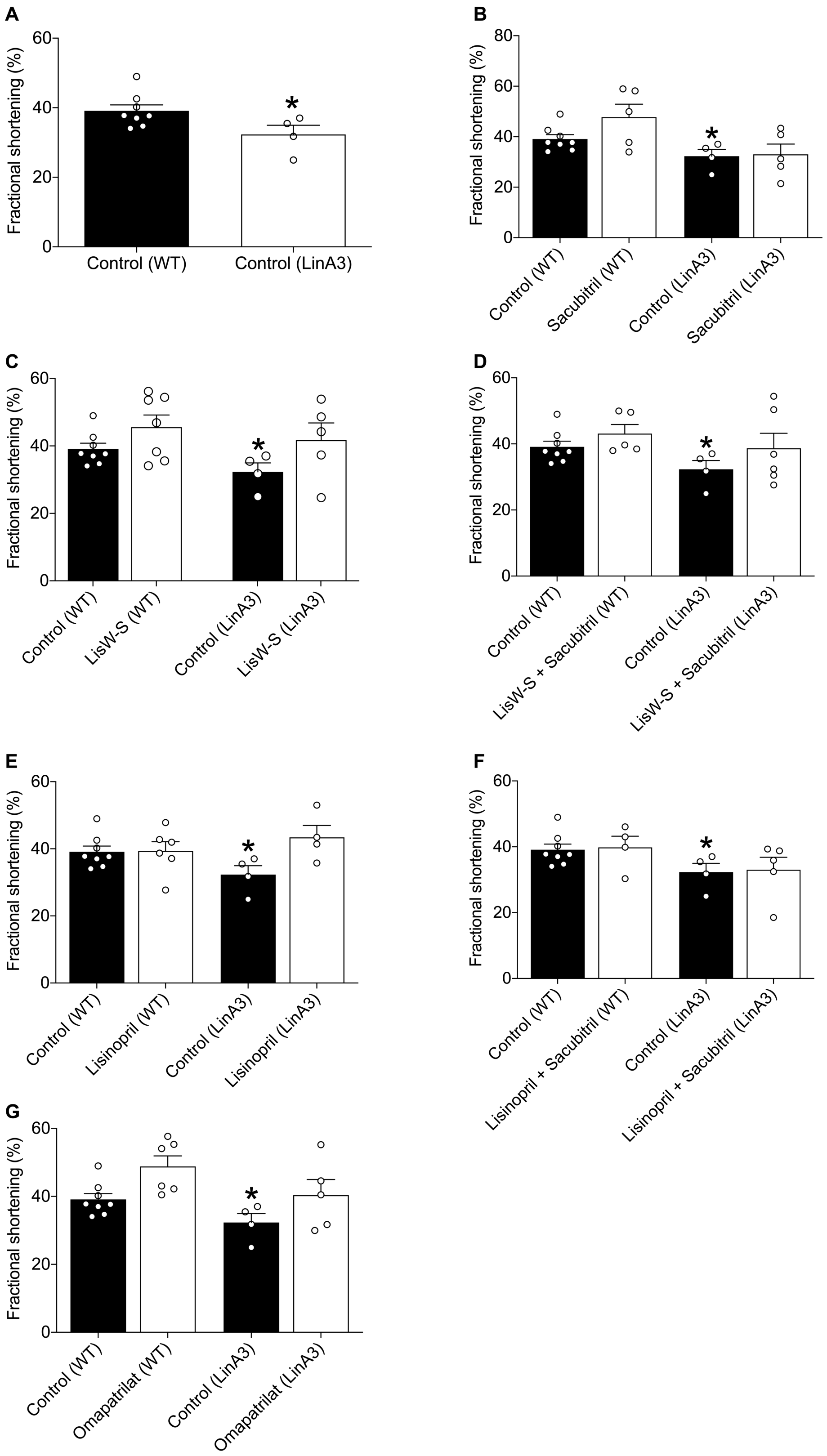

Figure S5. Inhibition of NEP and ACE system improves fractional shortening in hypertensive mice. Fractional shortening was measured by echocardiography in WT and LinA3 mice controls (A) and treated with sacubitril (B), lisW-S (C), lisW-S + sacubitril (D), lisinopril (E), lisinopril + sacubitril (F) and omapatrilat (G). Values express % of fractional shortening±SEM (n=4-8; One-way ANOVA with Dunnett post-test). P<0.05. *: *vs* WT control.

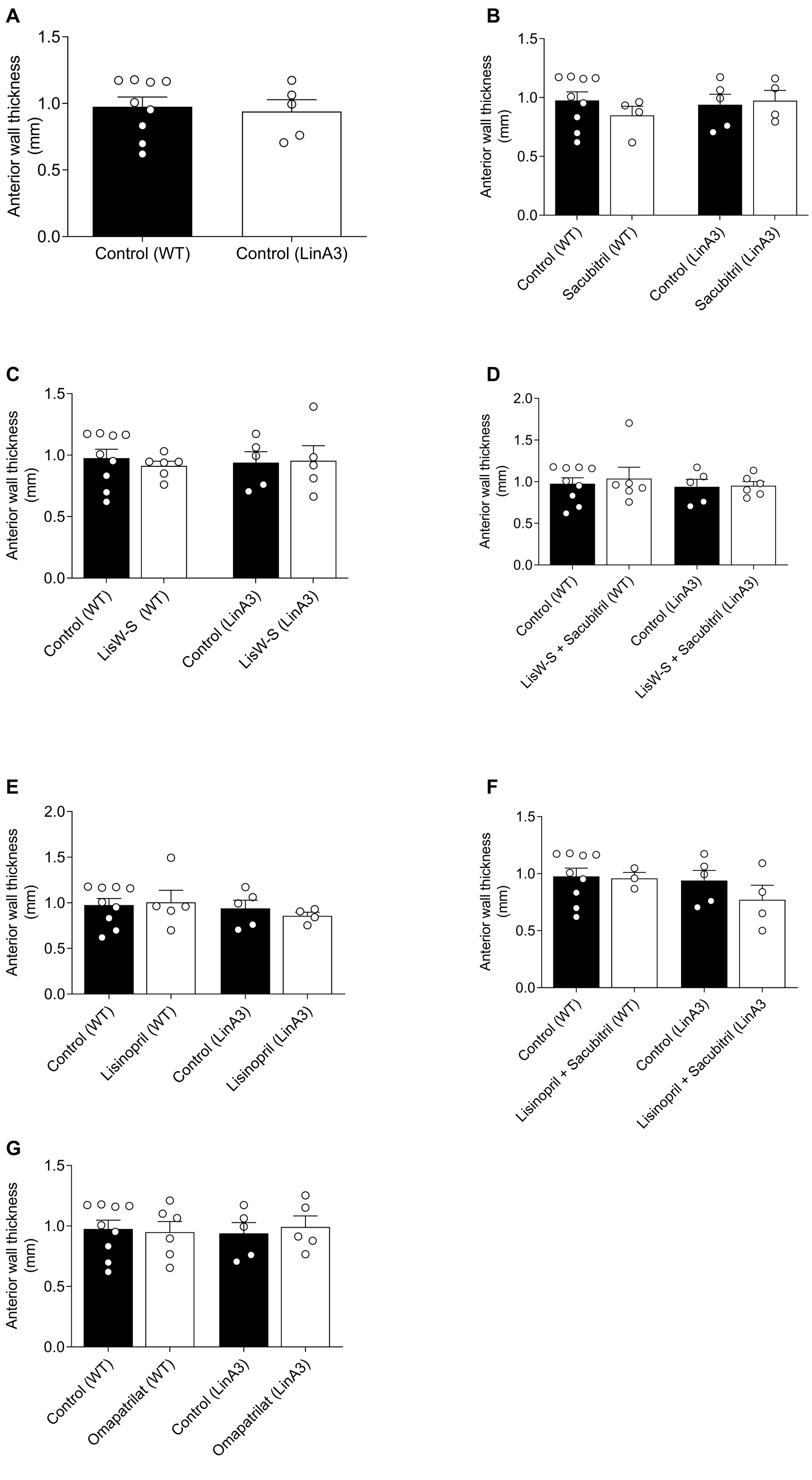

Figure S6. No changes are observed in anterior wall thickness in heart from hypertensive compared to WT mice. Anterior wall thickness was measured by echocardiography in WT and LinA3 mice controls (A) and treated with sacubitril (B), lisW-S (C), lisW-S + sacubitril (D), lisinopril (E), lisinopril + sacubitril (F) and omapatrilat (G). Values express anterior wall thickness (mm) ±SEM (n=3-9; two-way ANOVA with Bonferroni's post hoc test).

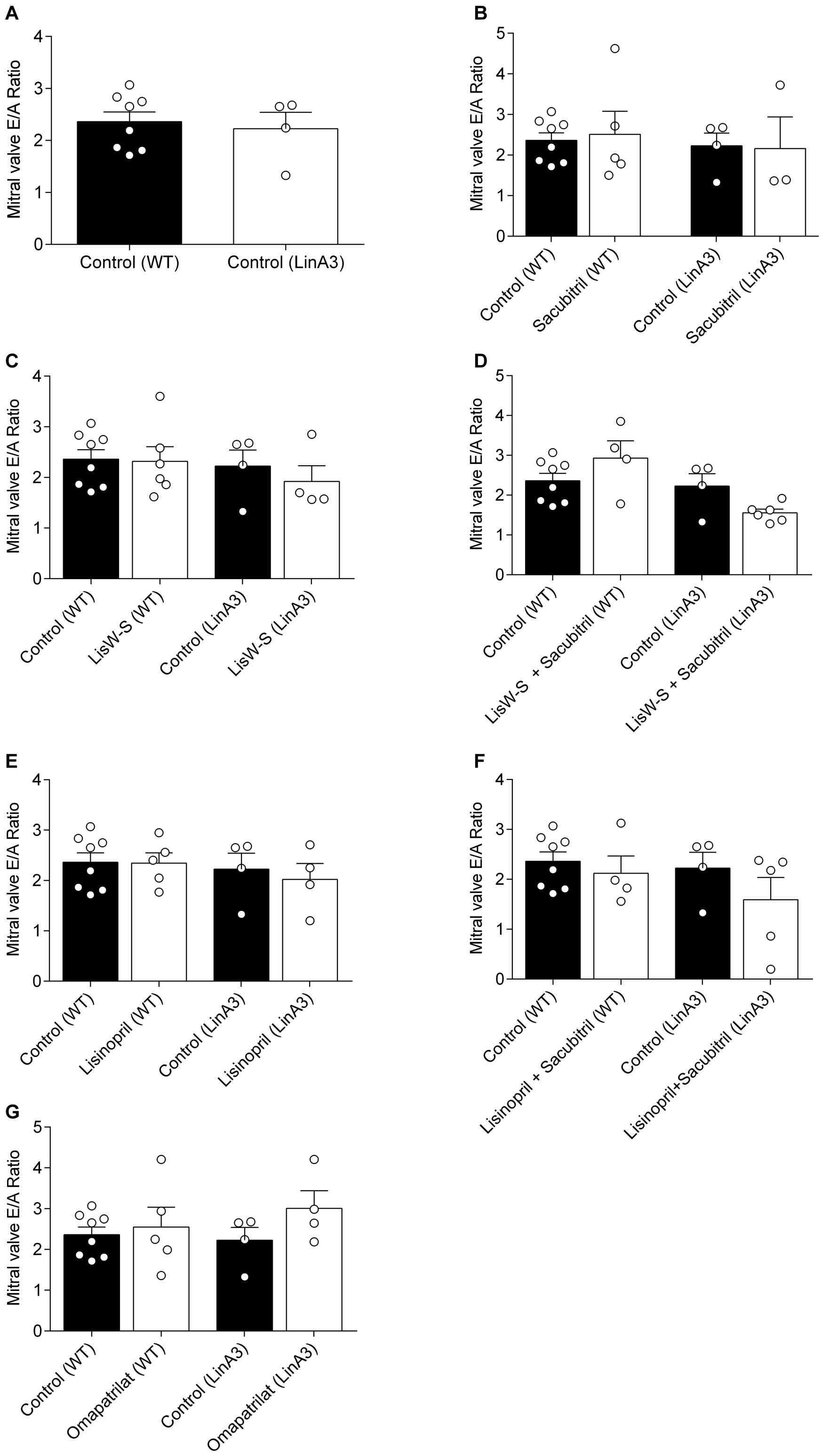

Figure S7. No differences are observed in the ratio of the early (E) to late (A) ventricular filling velocities in heart from hypertensive mice compared to WT mice. Mitral valve E/A ratio was measured by echocardiography in WT and LinA3 mice controls (A) and treated with sacubitril (B), lisW-S (C), lisW-S + sacubitril (D), lisinopril (E), lisinopril + sacubitril (F) and omapatrilat (G). Values express anterior wall thickness (mm) ±SEM (n=4-8; One-way ANOVA with Dunnett post-test).

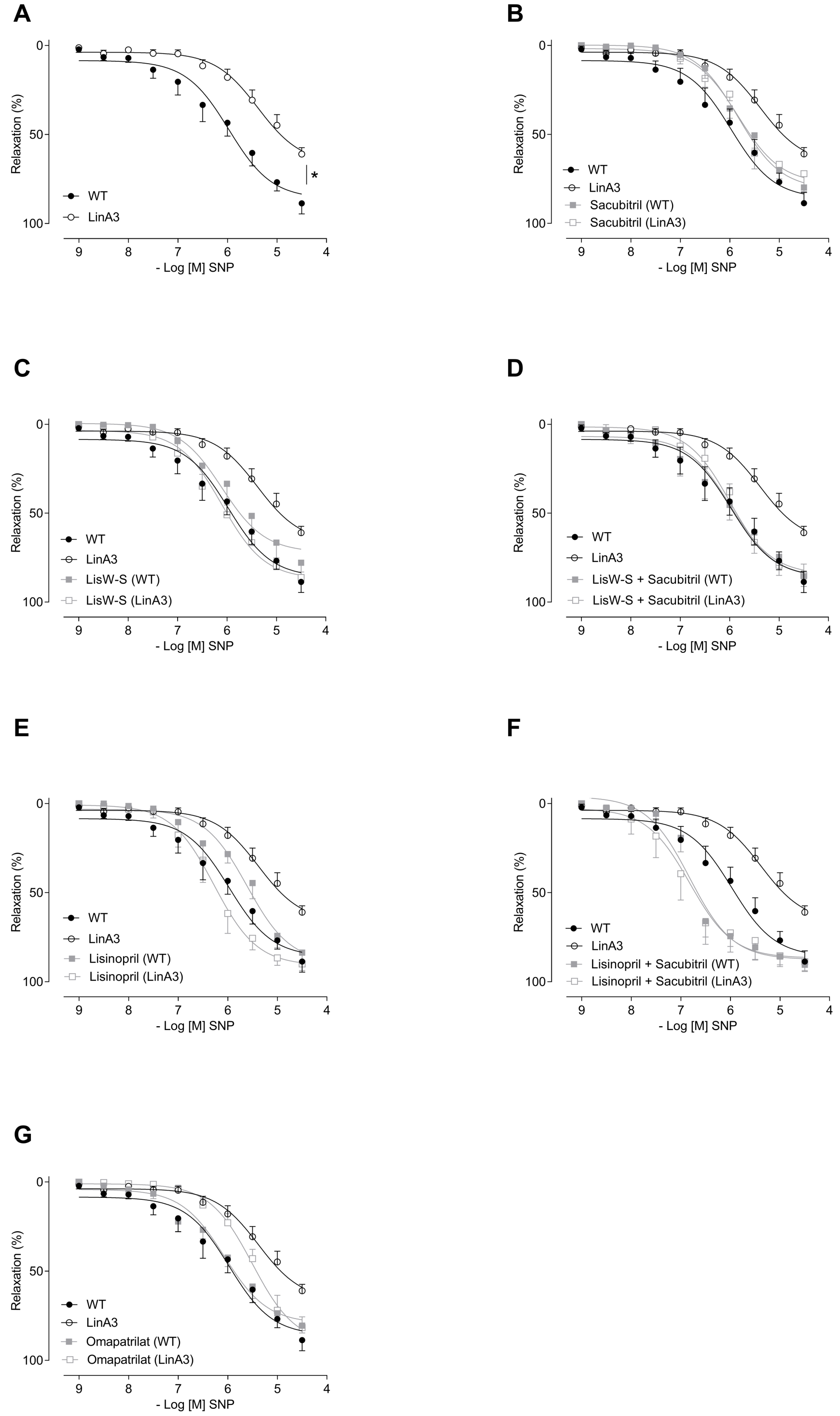

Figure S8. Inhibition of NEP and ACE system improves endothelial-independent relaxation in hypertensive mice. The concentration-response curves to SNP were performed in mesenteric arteries isolated from WT and LinA3 mice controls or treated with sacubitril (B), lisW-S (C), lisW-S + sacubitril (D), lisinopril (E), lisinopril + sacubitril (F) and omapatrilat (G) and mounted on wire myograph. SNP curves (relaxation) were expressed in % of relaxation compared to pre-constriction induced by U44619 and represented as mean ± SEM (n=5-6; two-way ANOVA with Bonferroni's post hoc test). P<0.05. * *vs* WT.

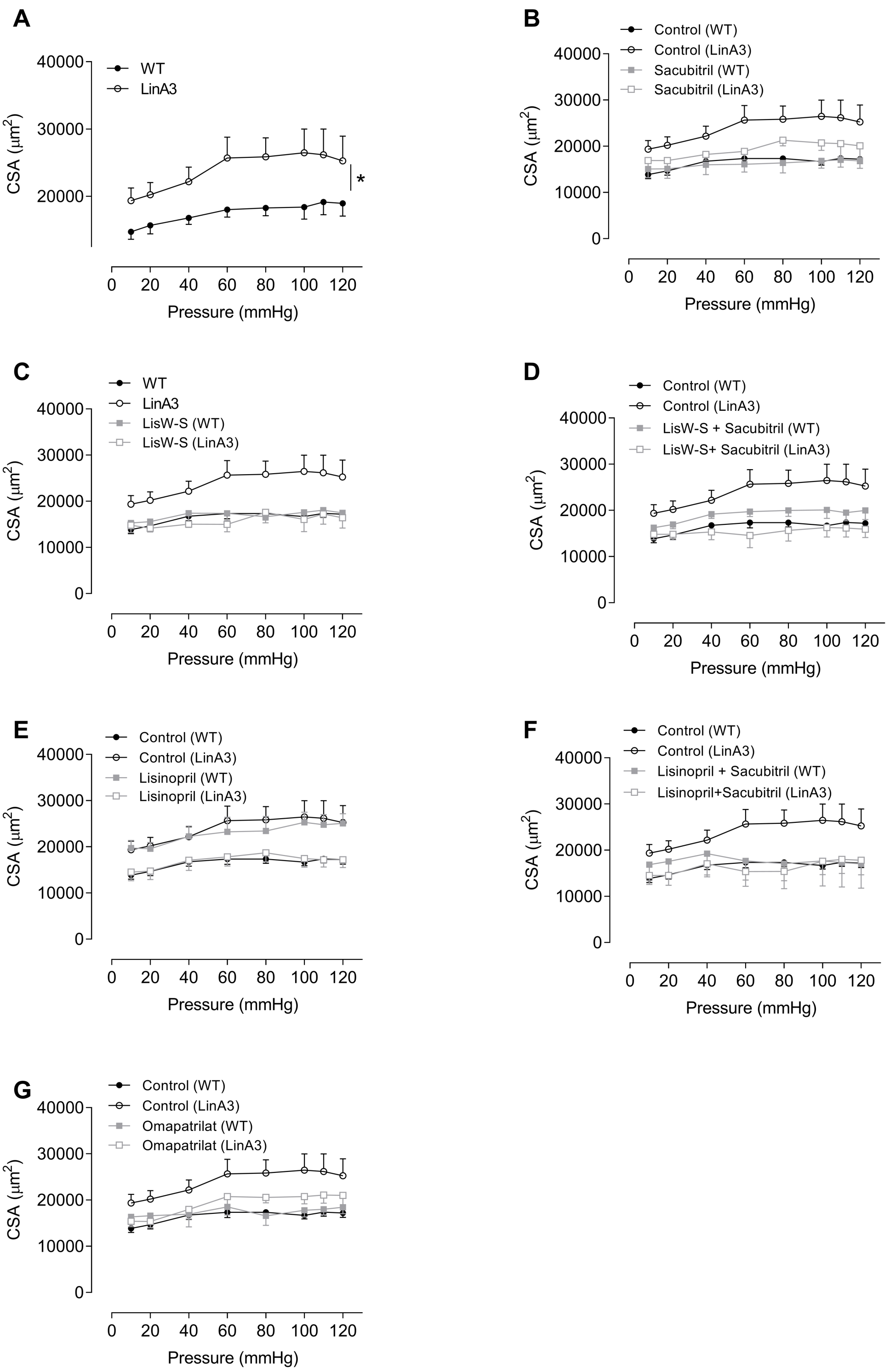

S9. Increased cross-sectional area in hypertensive mice is not observed in mice treated with inhibitor of C-domain of ACE combined with NEP inhibitor. Vascular structure was assessed in pressurized small arteries obtained from WT and LinA3 mice controls or treated with sacubitril (B), lisW-S (C), lisW-S + sacubitril (D), lisinopril (E), lisinopril + sacubitril (F) and omapatrilat (G). (n = 6; two-way ANOVA with Bonferroni's post hoc test). Results are presented as μm^2 ± SEM. *P < 0.05 *vs* control.

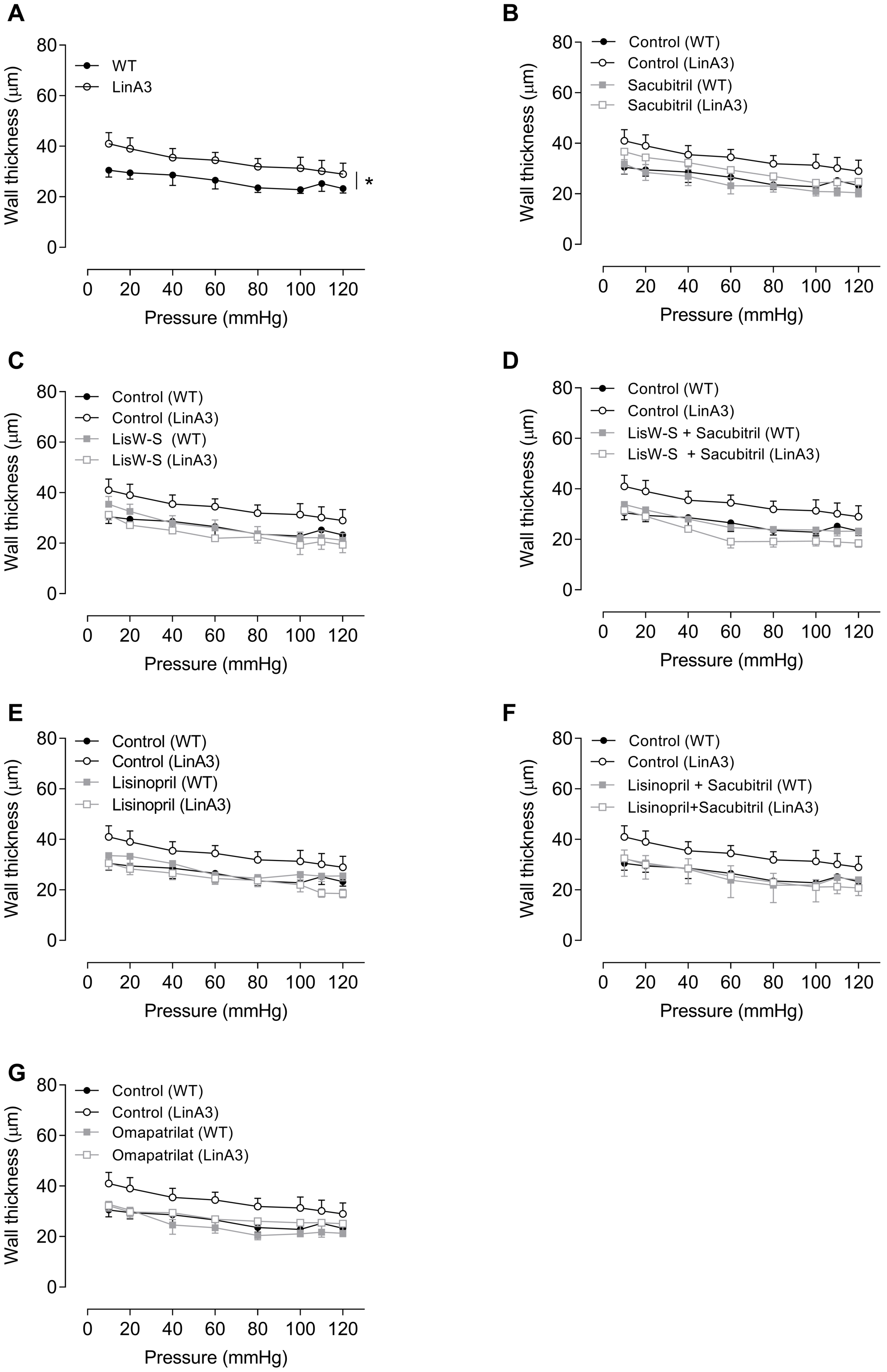

Figure S10. Increased wall thickness in hypertensive mice is not observed in mice treated with inhibitor of C-domain of ACE combined with NEP inhibitor. Vascular structure was assessed in pressurized small arteries obtained from WT and LinA3 mice controls or treated with sacubitril (B), lisW-S (C), lisW-S + sacubitril (D), lisinopril (E), lisinopril + sacubitril (F) and omapatrilat (G). Results are presented as $\mu m^2 \pm$ SEM (n=5-6; two-way ANOVA with Bonferroni's post hoc test). *$P < 0.05$ *vs* control.

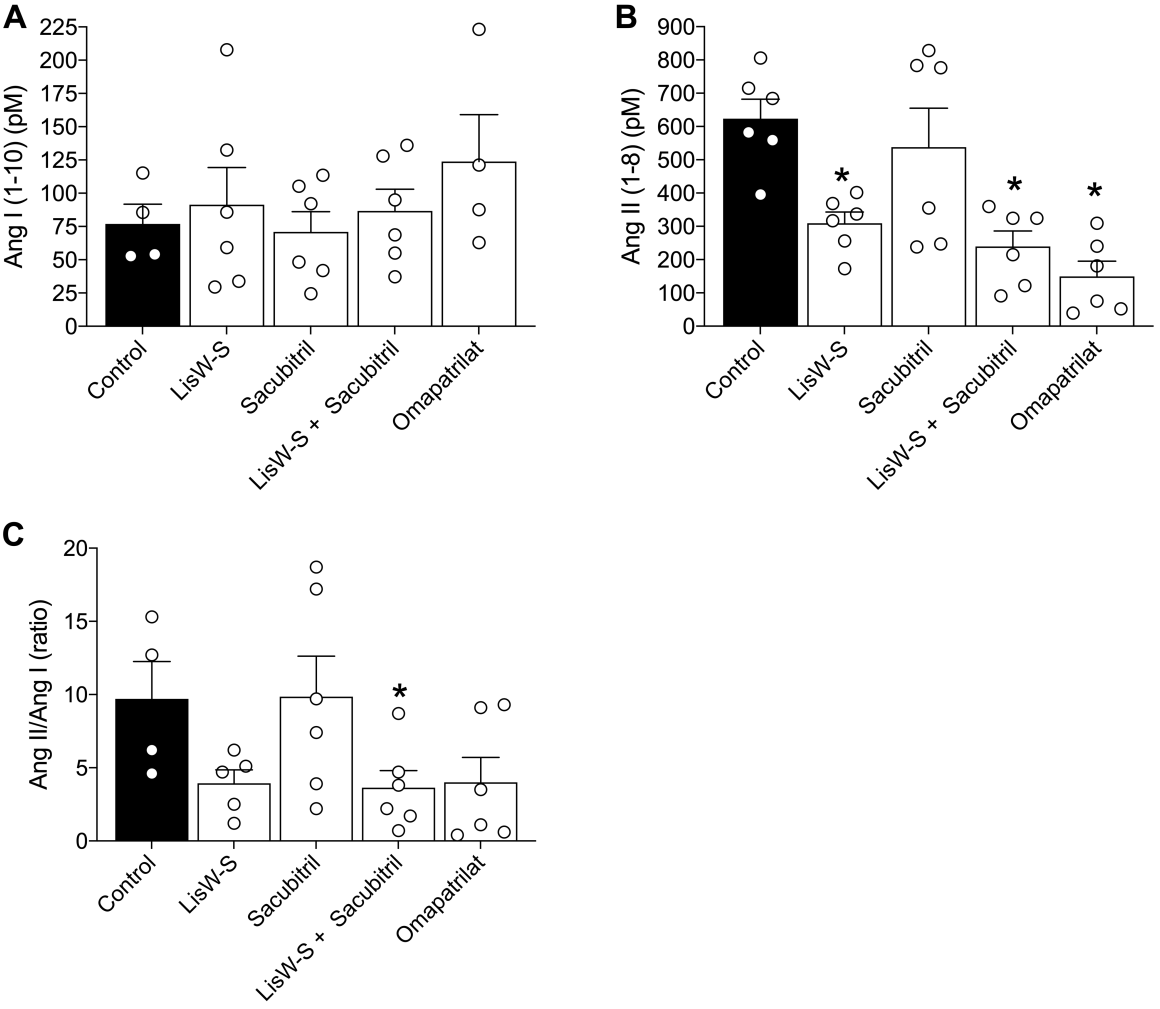

Figure S11. Effects of treatments on Angiotensin production in kidney in LinA3 mice. Angiotensin peptides were measured in kidney from LinA3 mice by LC-MS/MS (liquid chromatography – tandem MS) quantification. Peptides were measured in samples isolated from control, lisW-S, sacubitril alone or combined with lisW-S and omapatrilat-treated mice. Values express peptides levels and represent the mean±SEM (n=4-6; One-way ANOVA with Dunnett post-test). $P<0.05$. *: *vs* vehicle.

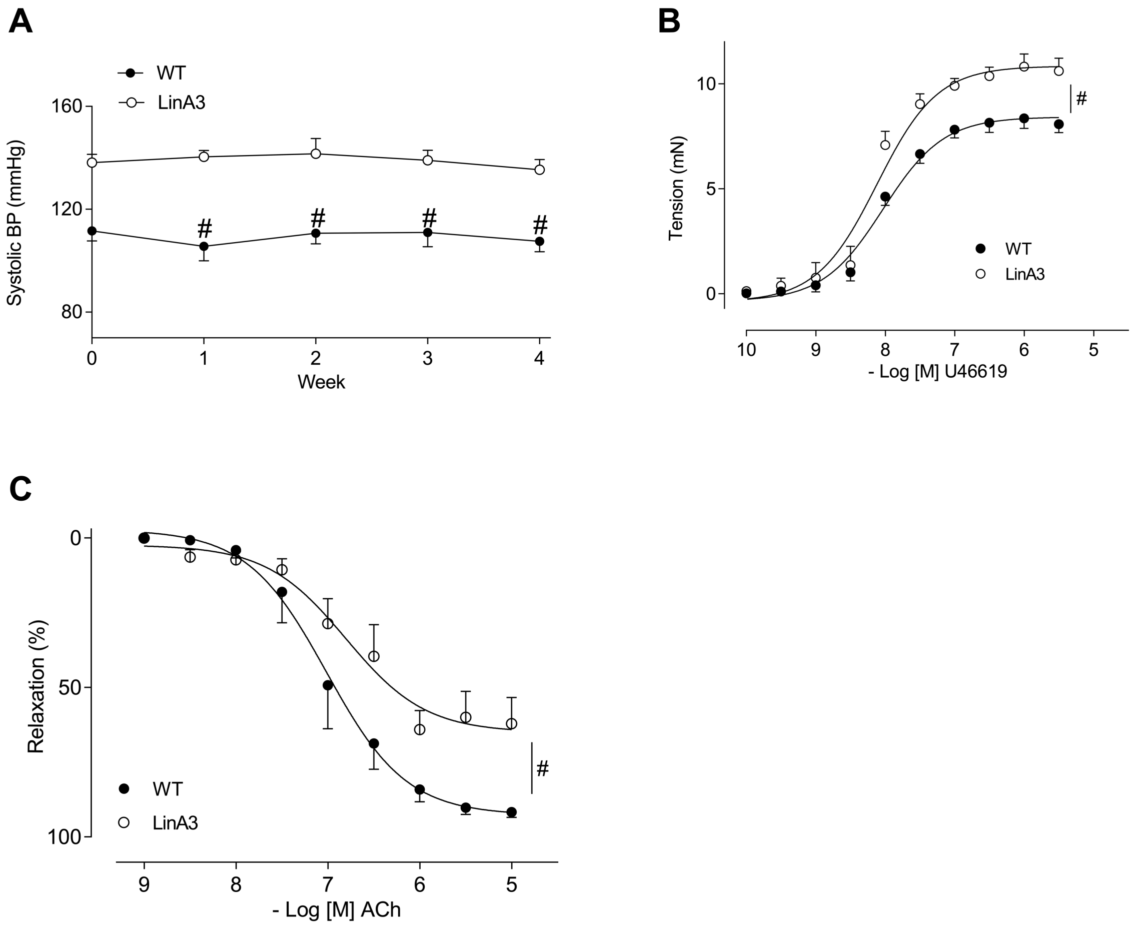

Figure S12: Systolic blood pressure and vascular contraction and endothelial-dependent relaxation of mesenteric arteries isolated from WT and LinA3 mice. Blood pressure was measured by tail cuff for 4 weeks and 1 week before starting the treatment (A). Concentration-response curves to U46619 (B) and ACh (C) were performed in mesenteric arteries isolated from WT and LinA3 mice. Data are mean±SEM (n=6; two-way ANOVA with Bonferroni's post hoc test). $P<0.05$. *: *vs* LinA3; $^{\#}$: *vs* WT.

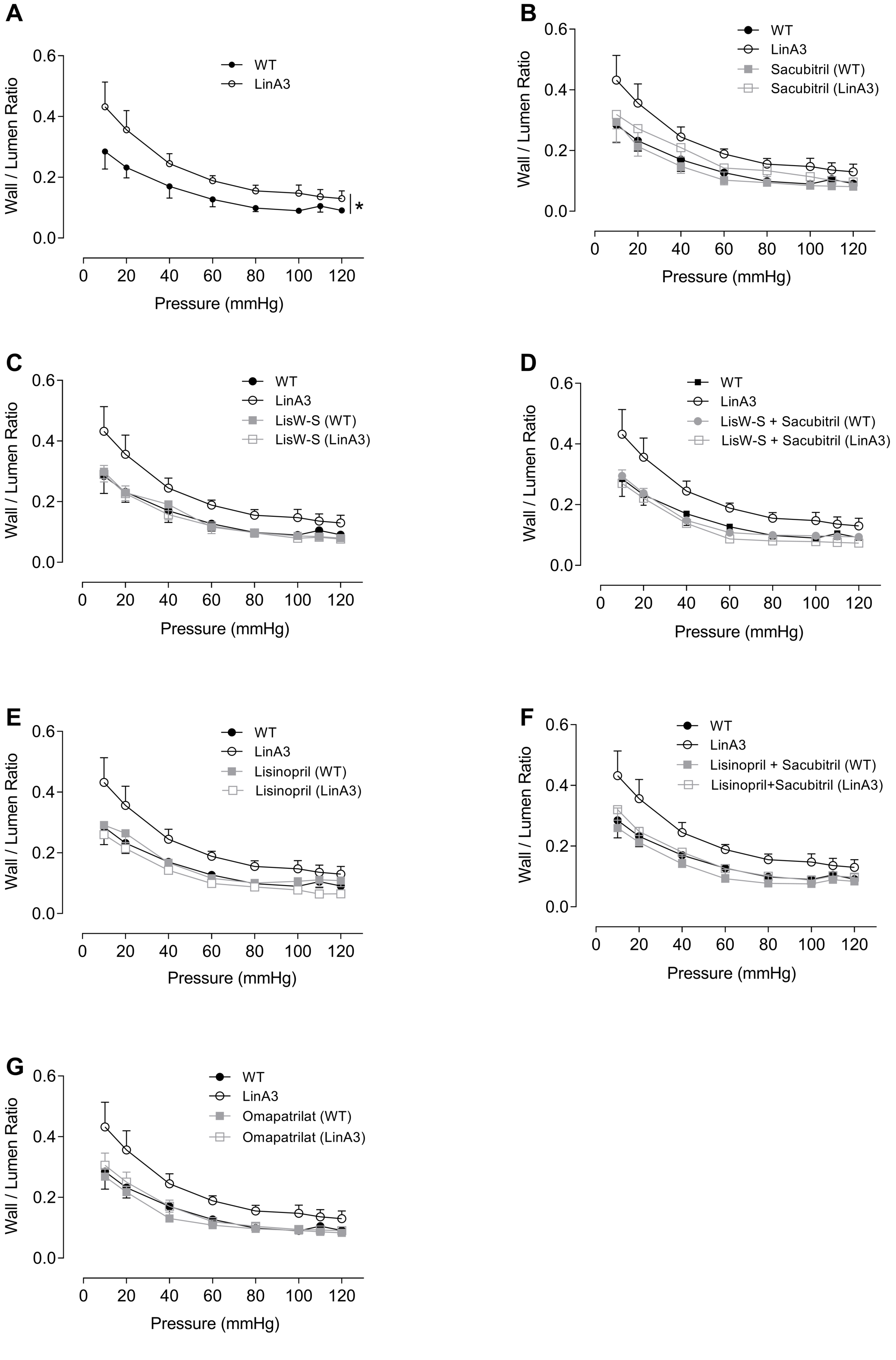

Figure S13. Treatment effects on wall/lumen ratio in hypertensive mice. Wall/lumen ratio was assessed in pressurized small arteries obtained from WT and LinA3 mice controls (A) or treated with sacubitril (B), lisW-S (C) lisW-S + sacubitril (D), lisinopril (E), lisinopril + sacubitril (F) and omapatrilat (G). Results are presented as μm ± SEM (n=5-6; two-way ANOVA with Bonferroni's post hoc test). P<0.05. * *vs* WT. # *vs* LinA3.

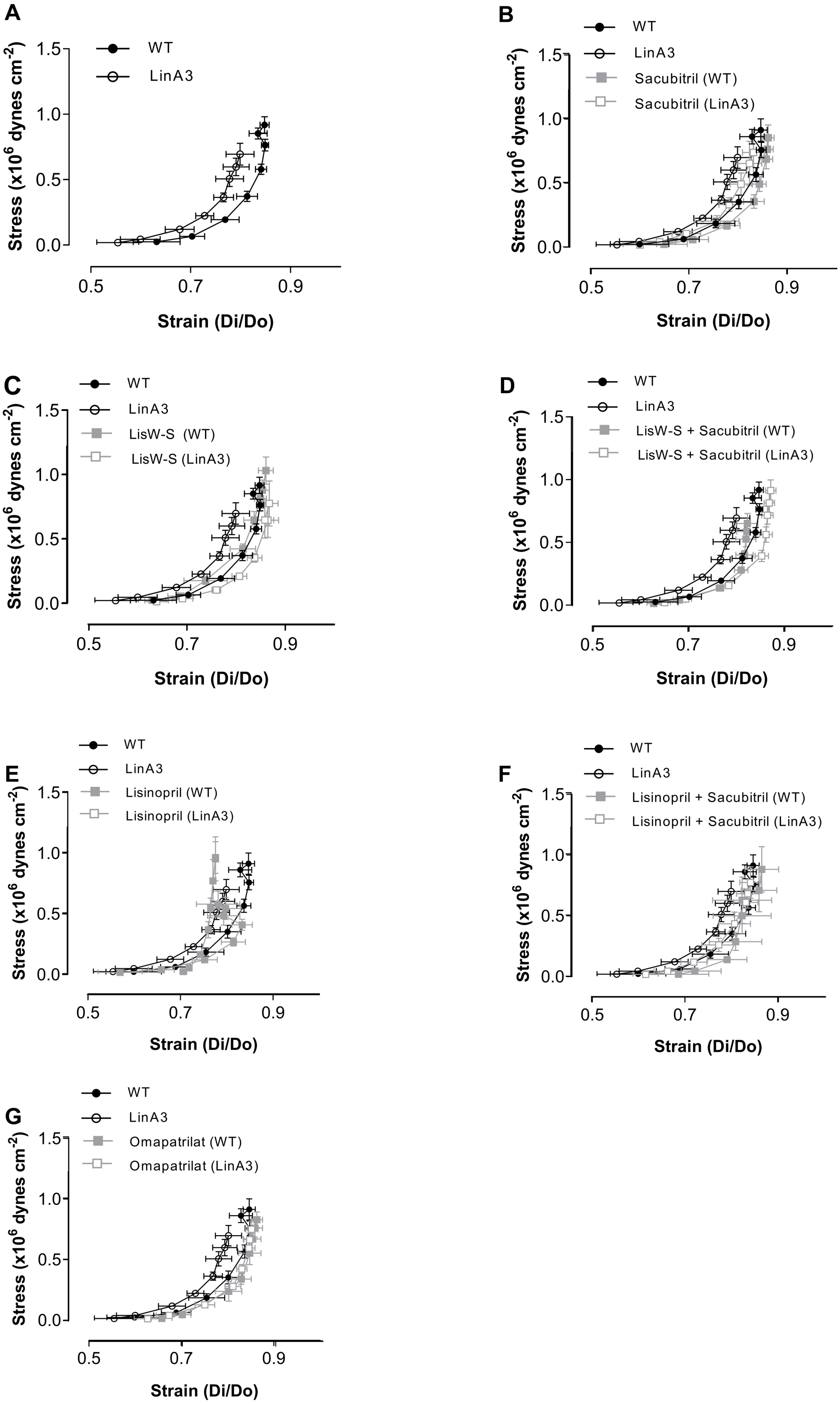

Figure S14. Mechanical properties in treated hypertensive mice. Stress-strain was assessed in pressurized small arteries obtained from WT and LinA3 controls (A) or treated with sacubitril (B), lisW-S (C), lisW-S + sacubitril (D), lisinopril (E), lisinopril + sacubitril (F) and omapatrilat (G). (n = 6). Results are presented as x10^6 dynes cm^{-2} ± SEM.
