# Supplementary figures and images for: Selective Inhibition of the C-Domain of ACE (Angiotensin-Converting Enzyme) Combined With Inhibition of NEP (Neprilysin): A Potential New Therapy for Hypertension
Source: Hypertension. 2021 Jul 26;78(3):604–16. doi: 10.1161/HYPERTENSIONAHA.121.17041 (PMC8357049; doi:10.1161/HYPERTENSIONAHA.121.17041)

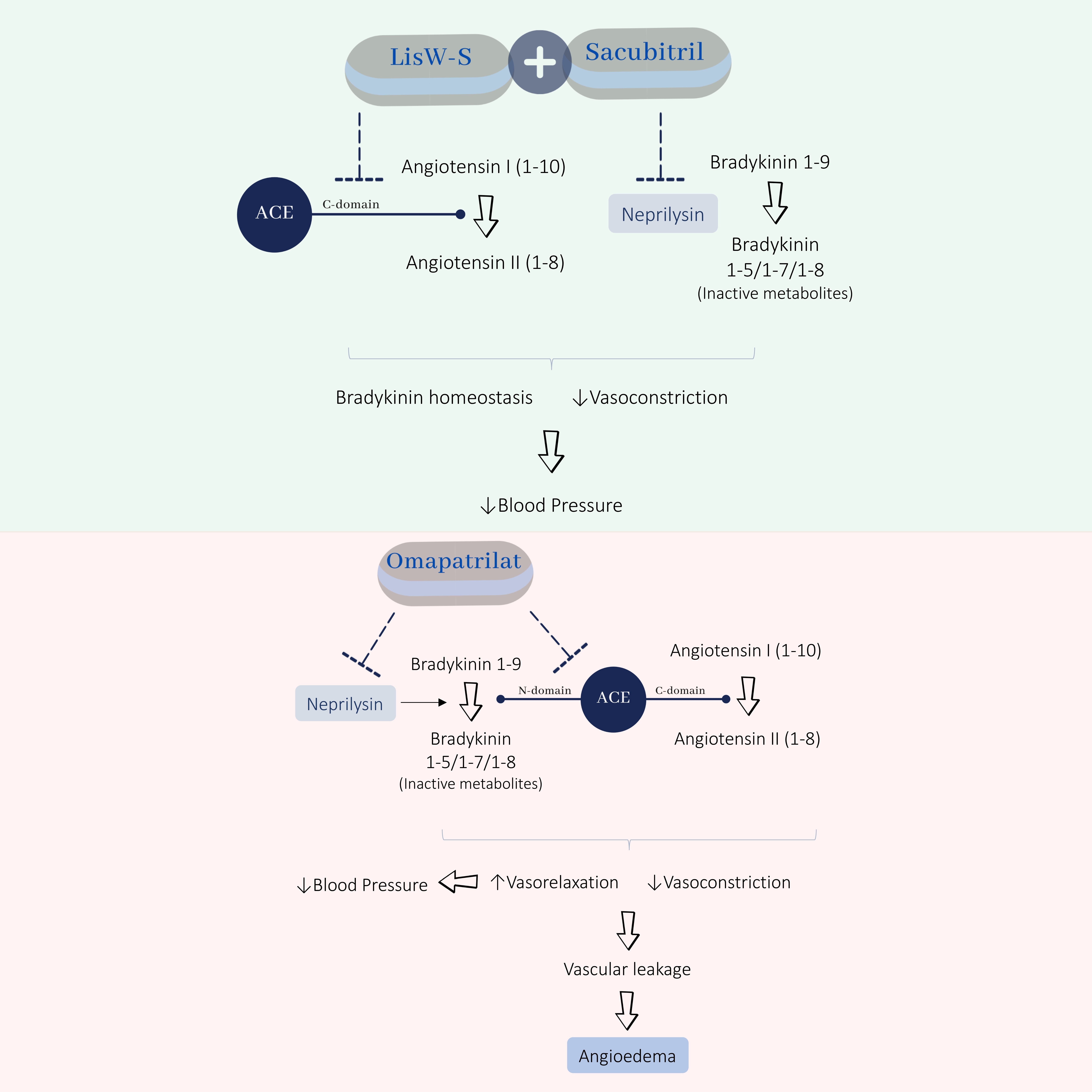

Supplement: Supplementary file 2 [file hyp-78-604-s002.jpg]
